# Usability and quality evaluation of the World Health Organization SkinNTDs app among frontline health workers in Cameroon: A mixed methods study

**DOI:** 10.1371/journal.pntd.0013461

**Published:** 2025-09-10

**Authors:** Henri Claude Moungui, Paul Tonkoung Iyawa, Hugues Nana-Djeunga, Jose Antonio Ruiz-Postigo, Carme Carrion

**Affiliations:** 1 Universitat Oberta de Catalunya, Barcelona, Spain; 2 Higher Institute for Scientific and Medical Research, Yaounde, Cameroon; 3 Helen Keller International, Maroua, Cameroon; 4 Prevention, Treatment and Care Unit, Department of Control of Neglected Tropical Diseases, World Health Organization, Geneva, Switzerland; 5 eHealth Lab Research Group, eHealth Center and School of Health Sciences, Universitat Oberta de Catalunya, Barcelona, Spain; International Atomic Energy Agency, AUSTRIA

## Abstract

**Background:**

Originally adapted from a paper-based guide for skin-related neglected tropical diseases (NTDs), version 3.0.0 of the World Health Organization (WHO) SkinNTDs app aims to strengthen disease surveillance and frontline health worker capacity in NTD-endemic settings. Evidence on its usability in routine care remains limited.

**Objective:**

To assess the usability and perceived quality of the SkinNTDs app in a real-life setting.

**Methods:**

This mixed methods evaluation was conducted between April and September 2024 among frontline health workers in five regions of Cameroon. Data included online questionnaires, based on the user version of the Mobile Application Rating Scale (uMARS), completed by 180 participants, and focus group discussions with 214 participants. Analyses were performed using jamovi 2.6.13 for quantitative analyses, and NVivo 12 Plus for qualitative analyses.

**Results:**

Participants reported limited dermatology experience (46.1% untrained or unexperienced), and nearly half were trained to use the app (66.1%). The app received moderate overall quality (mean = 3.61/5), with functionality and information scoring highest (both 3.69) and engagement lowest (3.50). Perceived impact was strong (3.88), and users were highly willing to recommend the app (3.96) but reluctant to pay (1.82). Prior app training to use the app was identified as the strongest predictor of higher quality ratings. Qualitative feedback highlighted critical needs: offline functionality (essential in low-connectivity areas), multilingual support, inclusion of darker skin tone images, and data-saving features. Digital barriers (e.g., data storage) and contextual adaptation were emphasized for effective implementation, alongside formal training integration.

**Conclusion:**

The app is a promising diagnostic support and educational tool, particularly when user training is provided. However, enhancements in engagement, cultural relevance (e.g., diverse imagery and local languages), offline utility, and reduced technical demands are critical for wider adoption. Scaling up adoption may be enhanced by integrating training modules into health system programs government endorsement, and addressing digital access barriers.

## Author summary

In this study, we evaluated the World Health Organization (WHO) SkinNTDs mobile app, an educational tool designed to help frontline health workers diagnose and manage skin-related neglected tropical diseases (NTDs). We worked with 180 healthcare professionals and nursing students in Cameroon to understand how usable and helpful they found the app. Using online questionnaires and group discussions, we discovered that while the app was rated moderately for overall quality (3.6 out of 5), users especially valued its role as a diagnostic support and learning tool.

Key feedback included requests for offline access (since internet connectivity is unreliable in rural Cameroon), and images showing darker skin tones to improve diagnosis accuracy. Health workers who received training on the app rated it significantly higher, underscoring the importance of hands-on support.

Our findings highlight that the app can empower frontline health workers in areas with limited access to dermatology specialist, but it must be adapted to local needs. We urge developers to prioritize offline features, and localized content, while policymakers should integrate the app into national health system, alongside providing devices and internet access to encourage adoption. Moving forward, we recommend testing the app in other regions and studying its long-term impact on disease detection and patient outcomes.

## Introduction

### Background

Mobile health (mHealth) is defined as “medical and public health practice supported by mobile devices, such as mobile phones, patient monitoring devices, personal digital assistants, and other wireless devices” [1]. By supporting training, supervision, and communication, digital health interventions have the potential to improve health worker performance [2]. For instance, a scoping review found that mHealth was a feasible digital health technology for lymphatic filariasis identification and mHealth, eHealth, and electronic health records improved the service access, outcomes, and monitoring of visceral leishmaniasis within community health systems [3].

Originally adapted from a paper guide for frontline health workers—defined as any health worker who directly provides service to a community [4]—on the signs and symptoms of neglected tropical diseases (NTDs) affecting the skin [5], version 3.0.0 of the World Health Organization (WHO) SkinNTDs app may enhance disease surveillance and contribute to the capacity building of frontline health workers in areas where NTDs are endemic [6,7]. Once a user opens the app, available without charge from Google Play and Apple Store [8,9], the welcome menu displays the functionalities of the app: ‘Signs & symptoms’ and ‘Diagnoses’, ‘How to manage’, ‘Glossary’ and ‘Skin NTDs Learning hub’. Examples of screenshots from Skin NTDs App are available on Internet [10,11].

Researchers are encouraged to assess the diagnostic accuracy of digital health tools and conduct further qualitative studies to inform end-user experience [12], and WHO has published guidelines for assessing digital health interventions and reporting these evaluations [13,14]. Considering these guidelines, the stage of maturity of the SkinNTDs app is the first and second stage. This means it seems appropriate to evaluate its feasibility and usability answering questions regarding how the app is used by end users, and how it fits into their workflow [4]. Various questionnaires are used for evaluating satisfaction, usability, acceptance, and quality outcomes of mHealth services. The top five of these, according to a recent review, include by order of citation the system usability scale – SUS, mobile application rating scale – MARS, post study system usability questionnaire, user mobile application rating scale – uMARS, and the technology acceptance model [15]. For example, a recent study to evaluate the usability and effectiveness of the eSkinHealth app for detecting and managing skin diseases in rural Côte d’Ivoire used the SUS tool [16]. The second top listed questionnaire, the MARS tool was developed to provide researchers, professionals, and clinicians with a brief tool for classifying and assessing the quality of mHealth apps [17]. However, training and expertise in mHealth and the relevant health field is required to administer it. This led to the development of a simpler, end user version of the MARS (uMARS) [18].

The first assessment of the WHO SkinNTDs app version 3.0.0 as a training tool, conducted in Ghana and Kenya [4], using the uMARS, concluded that the app demonstrates notable usability and user-friendliness and, with improvement, could make a crucial contribution to the capacity building of frontline health care workers dealing with skin NTDs. A more recent scoping review has recommended that developers consider adapting digital health tools to the diverse socio-cultural and technical environments in which skin NTDs are endemic [12]. Accordingly, comparable evaluations in various contexts are required in order to determine any additional context-specific experiences and validate these early findings. Thus, this study will assess the usability and user experience of the WHO SkinNTDs app as a capacity building tool in case identification and management among frontline health workers in Cameroon. As such, the study aimed to answer the following research questions: 1) How do users in Cameroon perceive the WHO SkinNTDs app? 2) How do users in Cameroon describe their current acceptance and use of WHO SkinNTDs app?

## Materials and methods

### Ethics statement

This study received two ethical approvals: from the Open University of Catalonia, Barcelona, Spain (CE22-TE29, signed on April 26, 2022); and from the National Ethics Committee for Health Research in Cameroon (N°2024/01/1620/CE/CNERSH/SP, signed on January 17, 2024). In addition, we requested and obtained administrative authorizations signed by regional public health delegates in the Far North and North regions.

All participants were provided with information about the study and it was explained that they could withdraw at any time without prejudice. A statement to this effect was included in the online questionnaire. Prior to recording focus group sessions, participants were informed that audio recordings were intended to ensure the research team accurately captured all discussions and would remain strictly confidential. Each participant provided oral informed consent and all data used for analysis were anonymized.

### Study design

Between April and September 2024, a mixed methods evaluation was conducted among frontline health workers in Cameroon. The study design was inspired by the protocol of a similar study implemented in Ghana and Kenya [19]. This mixed methods study addressed acceptability and usability of the WHO SkinNTDs app. A convergent mixed methods design was used, and it is a type of design in which qualitative and quantitative data are collected in parallel, analyzed separately, and then merged [20]. In this study, we used the uMARS questionnaire which is a simple tool that can be reliably used by end-users to assess the quality of mHealth apps. uMARS quantitative data were used to assess the quality of the app through a usability test by the participants. These data were complemented with data from open-ended questions added to uMARS online questionnaires. The qualitative data from focus groups and key informant questionnaires added further details on participants acceptability and usability of the app. The reason for collecting both quantitative and qualitative data was to fully capture participants’ perspectives.

### Study setting

Like many other low-and-middle income countries (LMICs), Cameroon has limited internet access in rural areas and varying levels of smartphone penetration, despite recent investment and improvement in the national digital infrastructure [21]. These factors are critical when assessing the feasibility and usability of mobile health applications.

**Fig 1:**
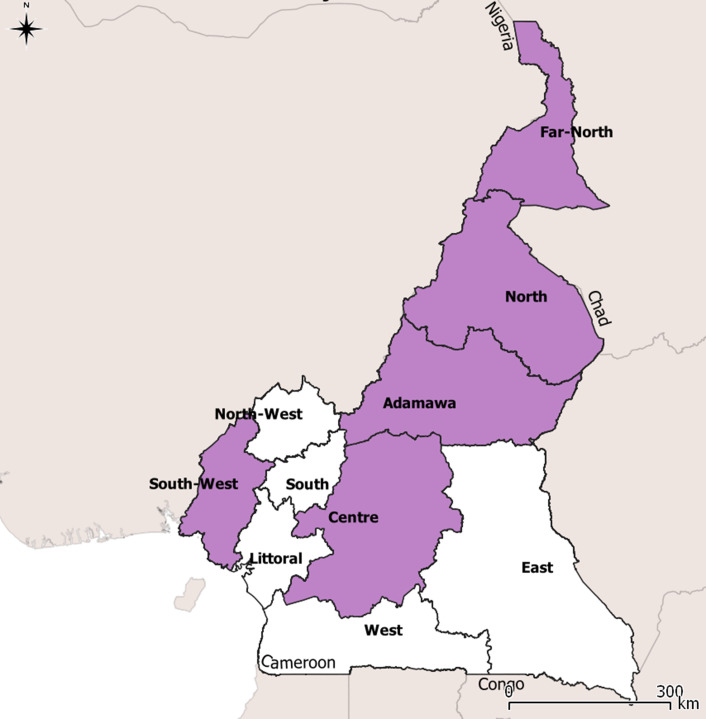
Regions in Cameroon where study participants were located. Map of Cameroon highlighting the regions where participants in the study were located.

Under the Ministry of Public Health (MOH), the country is divided into ten health regions administered by a regional delegation. Each region is then divided into health districts, and each district into health areas. Within the MOH, the national NTDs program is directed by a NTDs coordination unit, which collaborates with non-governmental organizations (NGOs), research institutions, and the national WHO office. We aimed to cover all ten health regions, including the North West and South West, the only English-speaking regions. Fig 1 shows the regions of Cameroon where the study participants are based. The map for Cameroon was downloaded from https://gadm.org/download_country.html version 4.1, under the https://gadm.org/license.html license, which allows for the free use of data for academic purposes including creating maps for research articles published in Open Access journals. The data were plotted onto that map using the QGIS software v3.0.0.

### Sample size calculation

To determine the necessary sample size, we followed the WHO recommendations in *Monitoring and evaluating digital health interventions* [13] that had also been applied in the Ghana and Kenya study [4]. As the SkinNTDs app is at the prototype stage, WHO suggests that, for usability studies, sample size should be between 10 and 100 individuals. Anticipating a 50% dropout rate due to this study being conducted on the internet, we opted for a minimum of 50 participants. For qualitative data collection like in the Ghana and Kenya study, we aimed to include a minimum of 10% of the overall sample size or continue data collection until reaching a point of information saturation.

### Eligibility criteria

Participants were eligible for the study if they met the following criteria:

**1.** **Health professionals or third-year nurse students:** Individuals working in public or private health facilities, or at levels above health facilities (e.g., health district services, regional delegations of public health, national NTDs program, and NGOs) or students in their third year of accredited graduate-level nurse training.
**2.** **Willing to access the app:** Willing to download and use the WHO SkinNTDs app prior to completing the survey.
**3.** **Willing to participate:** Willing to provide informed consent to participate in the study, including consent for focus group discussions and audio recording.

### Sampling strategy

To reach the target population, we employed a combination of non-probabilistic snowball sampling and purposive sampling. The chain of contacts began with a regional NTDs coordinator, who reached out to colleagues at the district level. District health staff then contacted colleagues working in health facilities. At each level, information was shared about the app, including download instructions and the link to the online questionnaire. A member of the research team actively facilitated this chain of communication.

### Recruitment and communication

Dissemination of the app to research participants was a prerequisite for enrollment. To facilitate communication and promote the app, we created a WhatsApp group. We progressively enrolled 57 individuals in the group after undergoing training sessions on the app by a research team member. By September 30, 2024, only 2 out of the 57 individuals had dropped out. Additionally, the research team shared information about the app and the survey questionnaire link in various existing WhatsApp groups maintained by MOH regional and district services. We also informed about the app through video presentations of the app and through face-to-face practical presentations.

### Data collection tools and procedures

Data were collected online via Google Forms using an English-translated version of the uMARS questionnaire. The uMARS is designed to objectively evaluate mHealth apps to ensure they provide quality health information, are user-friendly, reliable, and meet health program needs [18]. We enhanced the original questionnaire by adding variables that relate to user experience. These variables are defined in supporting information file S2 Appendix.

Before starting data collection, we pre-tested the questionnaire and shared it individually with targeted participants via WhatsApp or through WhatsApp groups.

### Qualitative data collection

Further qualitative data were collected through focus group discussions between participants selected via convenience sampling. We initially reached out to regional or district staff for information on when frontline health workers were expected to come together, then scheduled site visits, ensuring in advance that those in attendance had received details about the app and recommendations on its use. On the day of the focus group, we briefed the health workers and invited them to participate.

We conducted six focus group sessions: three in the North region and three in the Far North region. These regions were those primarily targeted for a field pilot for dissemination of the app in the country. Though all the ten regions of the country are endemic to NTDs, Far North region is the only one in the country hosting a WHO reference center for the management of leishmaniasis. A total of 214 individuals attended, 100 females and 114 males, with between 15 and 78 participants at each 60–90 minutes session. Detailed information about these sessions is provided in supporting information files S1 Appendix and S1 Table.

### Strategic level key informant interviews

At the last stage of the data collection process, we added a specific Google Forms questionnaire to capture additional insights from key informants at the strategic level of the national NTDs program. This enabled us to gather expert perspectives on the usability of the app and its possible integration into the NTDs program. These questions are provided in supporting information file S2 Appendix.

### Quantitative data analysis

All items on the uMARS from the online questionnaire responses were re-coded on a 5-point scale (1 = inadequate, 2 = poor, 3 = acceptable, 4 = good, and 5 = excellent) [18]. The mean score for each individual item on the subjective quality scale was calculated. These individual mean scores were then grouped to determine the mean score for each uMARS subdomain: *Engagement*, *Functionality*, *Aesthetics*, and *Information*. Subsequently, the subdomain mean scores were averaged to compute the overall mean quality score. The same method was applied to calculate the subjective mean score and perceived mean score. We described data presenting minimum, maximum, and mean scores, and standard deviation (SD) as measure of variance.

We studied the relationship between uMARS domain mean scores and other survey variables, using the independent two-tailed Student’s t-test, ANOVA or chi-square tests. We further explored through a linear regression analysis which survey variables predicted the quality mean score. For these analyses, we checked data assumptions using Shapiro-Wilk tests for normality and Levene’s tests for equal variances. If these tests showed significant deviations (p < 0.05), we used non-parametric alternatives (Kruskal-Wallis or Mann-Whitney U tests for normality violations) or adjusted tests (Welch’s t-test for unequal variances). For group comparisons (e.g., professionals vs. students), we reported effect sizes to show practical importance through Cohen’s d for t-tests (with 95% confidence intervals), or Eta-squared (η²) for ANOVA, Epsilon-squared (ε²) or Rank-biserial correlation for non-parametric tests [22]. Statistical significance was set at p < 0.05, but we prioritized effect sizes and confidence intervals to interpret findings. Since we found no statistically significant differences in uMARS scores between professionals and students (all p > 0.05), we combined both groups’ data for our final analysis and presentation of results. Quantitative analyses were performed using the jamovi software version 2.6.13 [23].

### Qualitative data analysis

Suggestions and comments from individual online questionnaires and focus groups were analyzed using thematic analysis [24]. From the transcripts, we first identified major themes and sub-themes based on uMARS to complement the quantitative analysis results. We then identified additional themes and sub-themes not related to uMARS in order to fully capture participants’ perspectives. NVivo software version 12 Plus was used for qualitative analyses.

## Results

### Participant demographics

In total, 180 survey participants completed the online questionnaire, 101 of these were health professionals and 79 were third-year students at state-registered nurse training schools. Sociodemographic characteristics are shown in Table 1. Of the 180 survey participants, 66.1% (n = 119) were males, 58.3% (n = 105) were aged < 35 years, and 71.7% (n = 129) had self-reported medium knowledge about mobile technologies.

**Table 1:** Participant demographics in the World Health Organization SkinNTDs app quality assessment through the uMARS survey (n = 180) shown by frequencies (%).

Variable	Responses	Participants, n (%)
**Age (years)**		
(n = 180)	< 35	105 (58.3)
	35–44	40 (22.2)
	45 +	35 (19.4)
**Sex**		
(n = 180)	Male	119 (66.1)
	Female	61 (33.9)
**Region**		
(n = 134)	Adamawa	1 (0.7)
	Center	1 (0.7)
	Far North	56 (41.8)
	North	75 (56.0)
	South West	1 (0.7)
**Participant type**		
(n = 180)	Professional	101 (56.1)
	Student	79 (43.9)
**Type of working institution**		
(n = 180)	Health facility (Public, Private, Parapublic)	78 (43.3)
	Above site facility	20 (11.1)
	Nurse training school	82 (45.6)
**Work context**		
(n = 180)	Rural	79 (43.9)
	Semi-rural	34 (18.9)
	Urban	67 (37.2)
**Trained in Skin NTDs diagnosis and management**		
(n = 178)	Yes	40 (22.5)
	No	138 (77.5)
**Dermatology experience**		
(n = 180)	Not trained and not experienced	83 (46.1)
	Not trained but experienced	86 (47.8)
	Trained or experienced	11 (6.1)
**Dealing with skin NTDs (cases/month)**		
(n = 180)	Rarely. < 1 case per month	103 (57.2)
	Occasionally. 1–3 cases per month	57 (31.7)
	Frequently. 4–6 cases per month	14 (7.8)
	Usually. > 6 cases per month	6 (3.3)
**Knowledge of mobile technology**		
(n = 180)	High	44 (24.4)
	Medium	129 (71.7)
	Low	7 (3.9)
**Source of awareness about the app**		
(n = 179)	Researcher	61 (34.1)
	Colleague	38 (21.2)
	Institution or School (Director, lecturer, schoolmate)	43 (24)
	Via App Store (Apple, Google)	37 (20.7)
**Received training to use the app**		
(n = 180)	Yes	61 (33.9)
	No	119 (66.1)

The majority of participants (77.5%, n = 138) reported having received training in the diagnosis and management of skin NTDs, while 66.1% (n = 119) stated they had been trained to use the WHO SkinNTDs app. However, nearly half of the participants lacked training or experience in dermatology. Additionally, 57.2% (n = 103) reported rarely encountering skin NTD cases, seeing fewer than one case per month. Participants were recruited from five of the country’s ten regions, with 43.9% (n = 79) working or studying in rural areas.

### uMars score

On a 5-point scale, survey participants scored an app quality mean score of 3.61 (SD 0.73), a subjective mean score of 3.31 (SD 0.65), and a perceived impact mean score of 3.88 (SD 0.81). Results of the uMARS analysis are summarized in Table 2.

**Table 2:** Results of the validated user version of the uMARS.

Domain	Definition (what it measures)	Mean (SD)	Min – Max
**App quality mean score**	**Measures the degree to which the app provides quality health information, is user-friendly, reliable, and meets health program needs**	3.61 (0.73)	1.09– 4.96
*Engagement* mean score	Measures how engaging and enjoyable the app is for users.	3.50 (0.83)	1.20– 5.00
*Functionality* mean score	Measures how well the app technically functions.	3.69 (0.92)	1.00– 5.00
*Aesthetics* mean score	Measures the visual appeal and design quality of the app.	3.59 (0.87)	1.00– 5.00
*Information* mean score	Measures the quality, accuracy, and relevance of app content.	3.69 (0.79)	1.17– 5.00
**App subjective mean score**	**Measures user satisfaction and willingness to recommend the app.**	3.31 (0.65)	1.50– 4.50
**App perceived impact mean score**	**Measures perceived impact of the app on knowledge and diagnostic efficiency.**	3.88 (0.81)	1.00– 5.00

Of the four uMARS subdomains (*Engagement*, *Functionality*, *Aesthetics*, and *Information*) of the quality domain, *Engagement* received the lowest rating (mean = 3.50, SD 0.83), while the highest rated were *Functionality* and *Information*, ex-aequo, with a mean score of 3.69 (SD 0.92 and 0.79 respectively). The SDs of domains and subdomains ranged between 0.65 and 0.92, indicating medium dispersion.

**Fig 2:**
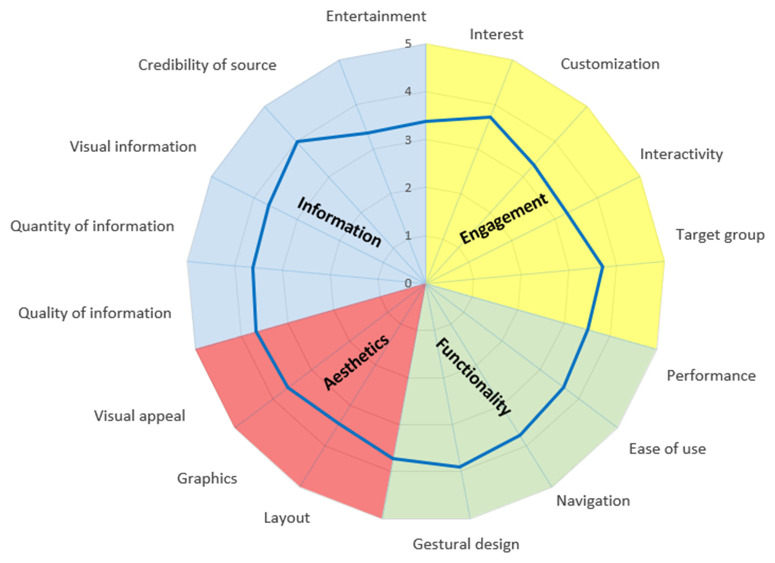
Radar chart evaluation of each app’s objective quality domain according to the user version of the Mobile Application Rating Scale (uMARS) in Cameroon.

Detailed results for each uMARS subdomain are presented in Table 3 and visually in Fig 2. In the *Subjective* subdomain, “willingness to pay for the app” received the lowest score (mean = 1.82, SD = 0.76), with a maximum score of 3. However, participants expressed a high likelihood of recommending the app to others, with a mean score of 3.96 (SD = 1.24). Furthermore, participants moderately recognized that the app can increase knowledge about sNTDs and strongly believe it will facilitate sNTDs diagnosis.

**Table 3:** Results of each uMARS subscale domain according to the study.

Subdomains	Mean (SD)	Min – Max
***Engagement* score*: Measures how engaging and enjoyable the app is for users.***		
Entertainment	3.37 (1.41)	1–5
Interest	3.73 (1.30)	1–5
Customization	3.35 (1.20)	1–5
Interactivity	3.29 (1.07)	1–5
Target group	3.71 (1.10)	1–5
***Functionality* score*: Measures how well the app functions technically.***		
Performance	3.52 (1.11)	1–5
Ease of use	3.59 (1.05)	1–5
Navigation	3.74 (1.20)	1–5
Gestural design	3.89 (0.99)	1–5
***Aesthetic* score*: Measures the visual appeal and design quality of the app.***		
Layout	3.71 (0.98)	1–5
Graphics	3.44 (1.03)	1–5
Visual appeal	3.61 (0.97)	1–5
***Information* score*: Measures the quality, accuracy, and relevance of app content.***		
Quality of information	3.68 (0.94)	1–5
Quantity of information	3.62 (0.98)	1–5
Visual information	3.65 (0.99)	1–5
Credibility of source	3.99 (1.01)	1–5
***Subjective items score: Measures user satisfaction and willingness to recommend the app***		
Would you recommend the app?	3.96 (1.24)	1–5
How many times do you use the app?	3.66 (1.03)	1–5
Would you pay for the app?	1.82 (0.76)	1–5
Overall (star) rating	3.81 (0.70)	1–5
***Perceived impact score: Measures the perceived impact of app on knowledge and diagnostic efficiency.***		
The app increases knowledge about sNTDs	3.54 (1.35)	1–5
The app will help for sNTDs diagnosis	4.20 (0.82)	1–5

### Analysis of other relevant questions added to the uMARS survey

Table 4 summarizes additional questions included in the uMARS survey. Notably, nearly all of the 180 participants supported the development of a desktop version of the app (n = 167, 92.8%), and 93.9% favored adding data-saving functionality for the recording of patient data. More than half of the participants (59.4%) supported translating the app, and language suggestions including French, English, Chinese, Spanish, Russian, German, Arabic and local languages in Cameroon. Approximately half of the participants were not in favor of adding internet-dependent functions, as they typically had little or no internet access on their phones.

**Table 4:** Other relevant questions added to the uMARS survey (n = 180).

Questions	Responses, n (%)
**How long since you downloaded the app?**	
Days (< 7 days)	139 (77.2)
Weeks (< 4 weeks)	12 (6.7)
1–2 Months	13 (7.2)
2–4 Months	8 (4.4)
> 4 months	8 (4.4)
**Did you receive training to use the app?**	
Yes	61 (33.9)
No	119 (66.1)
**Should the app be translated?**	
Yes	107 (59.4)
No	73 (40.6)
**Should sNTDs surveillance features be added?**	
Yes	167 (92.8)
No	13 (7.2)
**Should a data-saving function be added?**	
Yes	169 (93.9)
No	11 (6.1)
**Would you be interested in a desktop version?**	
Yes	163 (90.6)
No	17 (9.4)
**How often have you used the app since downloading?**	
Rarely	58 (32.2)
Occasionally	62 (34.4)
Frequently	44 (24.4)
Usually	16 (8.9)
**How strongly do you feel internet-dependent functions should be added?**	
Very uninterested. I hardly ever have internet connection on my phone	25 (13.9)
Uninterested. Most of the time I do not have access to the internet on my phone	53 (29.4)
Neutral. Sometimes I have internet on my phone	10 (5.6)
Interested. Most of the time I have internet on my phone	75 (41.7)
Very interested. I always have internet on my phone	17 (9.4)

We performed statistical analyses to check associations between the uMARS quality mean scores and the main demographic variables of study participants. The null hypothesis assumed no difference in the mean quality score across these variables. Our analysis showed that participants trained to use the app rated app quality 0.29 points higher than those untrained (mean = 3.80 vs. 3.51, mean difference = 0.291, 95% CI [0.291, Inf [, p = 0.005), with a small-to-medium effect size (Cohen’s d = 0.405). Moreover, participants who supported the addition of surveillance features rated the app 0.68 points higher than those who did not (mean = 3.66 vs. 2.98, mean difference = 0.686, 95% CI [0.172, Inf [, p = 0.017), medium-to-large effect size (Cohen’s d = 0.787). Significant differences were also observed in mean scores based on participant opinions on adding internet-dependent functions (χ^2^ = 19.0, p < 0.001, ε^2^ = 0.106): Participants that always have internet on the phone would rate the app’s quality 0.9 points more than those who hardly never have internet connection on their phone. All other comparisons showed no statistically significant differences including association between type of participants (professionals and students) vs quality mean score. Outputs of these analyses are detailed in supporting information file S3 Appendix.

As reported in supporting information file S3 Appendix, we further investigated if other uMARS scores differed between professionals vs students. Our investigation revealed that professionals (n = 101) scored the app’s perceived impact significantly higher than students (n = 79) (mean = 4.2 vs 3.4, mean difference = 0.833, 95%CI [0.102; Inf[, p < 0.001), with a large-to-very large effect size (Cohen’s d = 1.210), and app’s subjective subdomain than students (mean = 3.4 vs 3.2, mean difference = 0.833, 95%CI [0.664; Inf[, p < 0.001), with a medium-to-large effect size (Cohen’s d = 0.341). The tests showed no significant differences in engagement, functionality, aesthetics or information mean scores (all p > 0.15).

We then checked which variables influenced the differences in the subjective and perceived impact mean scores between professionals and students. We found that these differences were mainly driven by their overall star rating of the app (mean = 4.0 for professionals vs 3.6 for students, mean difference = 0.443, 95%CI [0.256; Inf[, p < 0.001, Cohen’s d = 0.624) and their perception that the app is likely to help as diagnostic support tool (mean = 4.2 vs 2.7, mean difference = 1.543, 95%CI [1.243; Inf[, p < 0.001, Cohen’s d = 1.340).

Finally, we also found that professionals were significantly more likely to be app-trained (80% vs 54%, χ² = 8.57, p < 0.01) but less likely to have formal Skin NTDs training (71% vs 86%, χ² = 5.96, p = 0.015). Students showed stronger rejection of a desktop version (15% vs 5%, χ² = 5.43, p = 0.02), suggesting mobile-first preferences.

### Regression analysis

We started with an initial multiple linear regression model with only individual variables significantly associated with quality mean score (Training to use the app, Preference for adding surveillance features, Preference for adding internet-dependent functions). That model explained 23.1% of the variance in the app quality mean score (R² = 0.231, adjusted R² = 0.204), indicating low explanatory power:

- **Training to use the app**: Participants who received training rated app quality significantly higher than those who did not (β = -0.389, SE = 0.105, p < 0.001).
- **Preference for adding surveillance features**: Participants in favor of adding surveillance features rated the app significantly higher (β = -0.726, SE = 0.188, p < 0.001).
- **Preference for adding internet-dependent functions**: Participants with consistent internet access rated the app significantly higher than those with limited internet access (β = 0.909, SE = 0.207, p < 0.001).

For detailed results of the regression analysis, refer to supporting information file S4 Appendix.

When these predictors were included in a multivariable regression alongside other survey variables not used in calculating the mean quality score, only training to use the app remained statistically significant (F = 5.25, p = 0.023), suggesting it is the most consistent predictor of the app’s mean quality score. The non-significant variables (surveillance features, internet dependency perceptions) may reflect overlapping effects or context-specific influences.

In a second linear regression analysis, we examined the statistical association between the app quality mean score and independent variables not included in its calculation. That model identified five significant predictors that explained 45% of app quality variance (R² = 0.450, adjusted R² = 0.430, p < 0.001) indicating a moderate explanatory power. With that new model, perceived knowledge improvement (β = 0.29, p < 0.001) and willingness to recommend (β = 0.17, p < 0.001) were the strongest predictors of app quality ratings alongside the intention to use the app frequently in the next 12 months (β = 0.12, p = 0.012). Formal dermatology training/experience increased ratings by 0.47 points (p = 0.010). Surprisingly, trained users rated quality 0.20 points lower than untrained users (p = 0.023). Assumptions of normality, independence, and multicollinearity were met.

### Results of qualitative data analysis

The suggestions and comments made in the questionnaires and focus groups were thematically analyzed. Frontline health workers in Cameroon expressed overall satisfaction with the WHO SkinNTDs app, appreciating its educational value, ease of use, and potential as a diagnostic support tool. As one participant noted, *“This app is a game-changer for diagnosing skin NTDs in remote areas.”*

#### Engagement and learning.

The app was recognized as a valuable learning resource:

***“The app is a great tool for learning about skin NTDs, especially for diseases we don’t see often.”***

Suggestions for enhancing engagement included interactive features:

***“Adding quizzes or case studies would make the app more engaging and fun to use.”***

#### Functionality and usability.

While the app was described as user-friendly, concerns were raised for some users:

***“The app is easy to navigate, but some of my colleagues struggle with smartphones. A simpler design would help.”***

The need for offline access was emphasized:

***“The app needs to work offline. In rural areas, we often don’t have internet access.”***

Accessibility features were also recommended:

***“I wish I could adjust the font size. The text is too small for me to read comfortably.”***

#### Cultural and contextual relevance.

Participants stressed the need for local adaptation in both content and language:

***“We need more images of darker skin tones. Many of the current images don’t match what we see in our patients.”***

***“The app should be available in Hausa and Fulani. Not everyone understands English.”***

#### Training and capacity building.

Many highlighted the importance of ongoing training and accessible language:

***“Many health workers in rural areas don’t speak English. The app needs to be in local languages.”***

***“We need regular training sessions to keep up with new features and updates in the app.”***

#### Integration and support.

Users emphasized that the app’s success depends on system-level support:

***“The government should officially endorse this app and include it in national health programs.”***

***“The government should provide smartphones and internet access to health workers in rural areas.”***

#### Technical and practical challenges.

Device limitations and digital barriers remain key challenges:

***“The app takes up too much space on my phone. I can’t use it because I don’t have enough storage.”***

***“In rural areas, we often don’t have electricity to charge our phones. Solar chargers would help.”***

A broader list of major themes and sub-themes, aligned with the uMARS framework or beyond, along with the most illustrative quotes, is available in supporting information file S5 Appendix.

In summary, participants suggested such additional themes as training, integration into healthcare systems, dissemination challenges, and cultural considerations. They also emphasized the importance of local language support, government endorsement, and the need to address technical barriers like internet access and device affordability to ensure wider adoption and effective use of the app. These findings will now be addressed in more detail below.

### Training on skin NTDs and the app

As less than half survey participants reported having received training to use the app, or on the diagnostic, manifestation, and treatment aspects of various skin-related NTDs, they requested additional training and more detailed information on app utility. Some participants thought the app should recommend medications.

### Key features identified

Participants identified several essential app features that support diagnostic processes:

- **Signs and Symptoms module:** Valued for its clear listing of disease indicators and serving as an “entry point” for identifying possible skin NTDs. Participants found it useful for both learning and practical reference during consultations.
- **Diagnosis module:** Highlighted as indispensable, particularly for its visual aids (images), which reduce diagnostic uncertainty by enabling clinicians to compare real cases with reference pictures.
- **Management option:** While less frequently referred to, this feature was appreciated for providing therapeutic guidance and follow-up suggestions.

Participants questioned the relevance of the ‘Global Index’ module and suggested a focus on regional statistics with regularly updated data would better address local practitioner needs.

### User feedback on app quality and features

Participant suggestions on how to enhance the app quality and features focused on the following areas:

- Adding more conditions to the app.
- Enhancing image quality and interactivity.
- Enabling offline access.
- Incorporating video and color images.
- Improving translations and customization options.

Participants also emphasized the need for enhanced knowledge-sharing capabilities, and complementary in-person training on the app and skin disease management. A broader list of the most pertinent suggestions and comments is available in supporting information file S5 Appendix.

### Participant suggestions to enhance practicality and user-friendliness

Participants recommended the following enhancements to make the app more practical and user-friendly:

- **Contextualization:** Include more country-specific data on skin NTDs, locally relevant statistics, and images of different skin tones to better address community needs.
- **Enhanced search functionality and offline use:** A search function and offline capabilities were requested to improve access in areas with unreliable internet.
- **Refinements to the chatbot:** The chatbot was seen as potentially redundant but could be useful if improved to provide individual diagnostic support.
- **Training and glossary:** Expand the glossary with more accessible terms and create quick-reference training modules to ease app navigation for new users.
- **Visual design:** Include more images of people with darker skin tones to improve diagnostic accuracy.

### Integration within the health system

The app was seen as a promising support tool, particularly for remote locations lacking dermatological specialists. However, several challenges were identified:

- **Patient-clinician relationship:** Some healthcare workers expressed concerns that using the app during consultations could erode patient trust and therefore recommended discreet usage.
- **Potential over-reliance:** There were concerns the app could lead to dependency and possibly weaken diagnostic skills over time.
- **Technical and resource limitations:** Challenges included limited and unreliable access to smartphones, internet connectivity, and electricity in remote areas.

### Specific comments from strategic-level key informants on app integration

Strategic-level informants recognized a possible contribution of the app as a decision-support tool but highlighted some prerequisites for its adoption:

- Health workers must find the app useful.
- The app should be tested with experts and health workers to validate its effectiveness.
- The reliability of the data underpinning the app must be ensured.
- Offline functionality and contextualization (e.g., translating the app into major international and local languages and dialects) should be provided.

### Ideal users and contexts

Participants believed the app would primarily be used by frontline health personnel, including nurses, dermatologists, and community health workers. In rural settings, community health workers would benefit from using the app for triage and referral purposes. Contexts included consultations, especially for unfamiliar cases, and in field activities such as disease surveillance and mass drug administration campaigns.

### Recommended dissemination strategies

Participants suggested the following strategies to maximize app adoption:

- **In-person training sessions:** Regular seminars for healthcare providers, particularly in low-connectivity areas.
- **Digital marketing:** Sharing app download links in professional WhatsApp groups and community health networks.
- **App store distribution:** Regular updates to ensure users have access to the latest features.

### Implementation challenges and solutions

Key challenges and proposed solutions included:

- **App size and storage:** Reduce app size to enable lower storage requirements and faster downloads.
- **Device and connectivity support:** Provide devices with the app pre-installed and periodic internet data packages for remote facilities.
- **Supplementary training:** Offer additional training on dermatology and skin NTDs to improve app effectiveness.
- **Pilot testing:** Conduct pilot testing and present results to stakeholders for successful integration within health systems.

## Discussion

### Principal findings

The WHO SkinNTDs app received moderate usability and quality ratings, with an overall app quality mean score of 3.61/5, a subjective quality score of 3.31/5, and a perceived impact score of 3.88/5. These results indicate that the app moderately meets the needs of frontline health workers in Cameroon, particularly in diagnostic support and educational value. However, the engagement subdomain scored the lowest (mean = 3.50/5), suggesting a need for improvements in interactivity (e.g., allow the user to further interact with the app) and user engagement.

The sequential regression analyses identified key factors influencing frontline health workers’ perceptions of app quality. An initial model, limited to three variables identified through individual association tests, explained 23% of the variance in the mean quality score. This model showed that lack of training, opposition to adding surveillance features, and limited access to internet-dependent functions were associated with lower quality ratings.

In the final multivariable model, which incorporated these variables along with additional perceived usefulness and behavioral intention variables, model performance improved substantially, explaining 45% of the variance. In this adjusted model, the strongest positive predictors of app quality were the perceived potential of the app to improve knowledge, willingness to recommend the app, and expected frequent use. Training and dermatology experience moderated app ratings, with trained or experienced users providing more critical but informed ratings.

### Interpretation of findings

The significant association between training in use of the app and app quality scores could reflect the fact that training provides a more in-depth understanding of app features and thereby increases app adoption. Most participants declared they would recommend the app (mean: 3.9/5), but few were willing to pay for it themselves (mean: 1.8/5). This could reflect the need for government endorsement of the app and also the fact that users were aware that the app is developed by WHO which usually donates drugs and other health equipment rather than selling.

While highly rating the quality of app information (mean: ~ 3.7/5), users requested more localized content to address context-specific challenges and offline functionality for areas with limited internet access. Training in dermatology also significantly improved app quality scores though nearly half the participants lacked the foundational knowledge to fully utilize the app. This suggests the app is more effective when users have prior clinical knowledge and implies the app functions best as a supplemental tool for trained professionals rather than a standalone solution for novices.

Unexpectedly, as shown in supporting information file S3 Appendix, we found no difference in app quality mean score between health professionals and third-year nurse students (p = 0.186). This suggests that this subpopulation could be used as a proxy for frontline health workers in future studies. Similarly, there was no statistical difference in willingness to pay among Professionals vs Students. Willingness to pay has not further been explored but in future studies this should be considered.

Our findings suggest that perceived educational benefit and behavioral intentions are the strongest drivers of positive app ratings, rather than infrastructural concerns like internet access or surveillance features. Efforts to improve adoption should therefore focus on strengthening the app’s educational components, enhancing user engagement (e.g., the app allows us), and integrating it into structured training programs, while ensuring it remains accessible in low-connectivity environments.

### Comparison of findings with the Ghana and Kenya study

The results from Cameroon align with the study conducted in Ghana and Kenya, in which the app also received moderate usability scores [4]. However, the app received lower scores in Cameroon in other aspects when compared to the study in Kenya and Ghana: an app quality mean score of 3.61/5 (SD 0.73) vs 4.02/5 (SD 0.47), a subjective quality score of 3.31/5 (0.65) vs 3.83/5 (SD 0.61), and a perceived impact of 3.88/5 (SD 0.81) vs 4.47/5 (SD 0.56) respectively. This may reflect the higher knowledge of mobile technology among users in Ghana and Kenya (68% vs 24% in Cameroon). It is also possible these differences relate to participants in Ghana and Kenya responding directly with the research team through online interviews, which may have led to interviewer bias [25]. As in Ghana and Kenya, Cameroonian participants favored the inclusion of skin NTDs surveillance, the ability to include patient records in the app, and the availability of a computer version of the app.

Our results diverge with a lack of association between the app quality mean score and the variables examined in the previous study, and revealed additional associations that should be considered when implementing the app in other countries.

Finally, our findings align with previous studies, for instance, the positive perception of the app a valuable decision support tool [26].

### Contextualization of findings

Contextual factors are likely to have contributed to the lower usability scores observed in Cameroon compared to Ghana and Kenya. The study was primarily conducted in northern Cameroon, a region with limited internet access and healthcare workers who often lack formal dermatological training. This highlights the importance of offline functionality and localized content to enhance app usability in resource-limited settings. While the app might contribute to bridge gaps in dermatological expertise, particularly in rural areas with limited access to specialists, its effectiveness also depends on addressing technical barriers such as offline access and interactivity requirements, for example personalized content/feedback [27].

### Strengths and limitations of this study

This study has important strengths:

- The study employed a mixed methods approach, combining quantitative data from the uMARS questionnaire with qualitative insights from participant suggestions and focus group discussions, thereby providing a comprehensive assessment of app usability and user experience.
- By including a diverse sample of healthcare workers, including both professionals and students, the study provides a broad perspective on the app.

However, this study has several limitations that warrant caution when interpreting findings:

- **Regional limitations in study representation:** The research was mainly conducted in the northern regions of Cameroon, which may limit the generalizability of results to the entire country. In addition, North and Far North regions share similar contexts in terms of healthcare burden, services and digital accessibility. Data from the southern regions, with their distinct socioeconomic contexts, could have provided different perspectives. Additionally, only three respondents from the two anglophone regions completed the online questionnaire, and no focus groups were conducted in these areas. Therefore, while the findings are somewhat representative of the eight francophone regions, their extension to the anglophone regions is limited.
- **Sampling bias**: The study relied on non-probabilistic snowball sampling, purposive sampling and convenience sampling. This may have introduced selection bias.
- **Deviation from focus group size guidelines:** We did not strictly follow guidelines on the optimal number of participants per focus group [28]. For example, in sessions involving state-registered nurse students, class sizes exceeded 20, with all students allowed to voluntarily remain in the room to avoid frustration. However, participants were fully informed of their right to withdraw or choose not to interact. In these cases, we did not systematically track the number of individuals who actively participated in the discussions.
- **Inconsistent compliance with app usage duration:** There is no way to ensure that online questionnaire respondents spent at least five days exploring the app before responding, as was stipulated in the protocol. However, since the original uMARS publication [18] recommends only 10 minutes of app testing, we believe the risk to the validity of our findings to be minimal.
- **Absence of response validation from participants:** We did not ask participants to validate their responses, which may have been particularly useful in relation to those presented within focus groups and to open-ended questions in the questionnaires. Among other issues, this would have been an opportunity to explore suggestions that the app be translated into other languages such as German, Spanish, Arabic, Russian, and Chinese, as well as local languages such as Fulani, and Ewondo. The assumption is that a participant would principally want the app translated into a language they most frequently use in daily life, yet the majority of participants were French speakers.
- **Use of a non-validated French translation of uMARS:** As almost all study participants were French speakers, we used our own translation of the English uMARS questionnaire. Since the conclusion of our fieldwork, a validated French version of the tool (uMARS-F) has been published [29].
- **Limitations in user experience insights due to lack of participant observation:** The study did not include participant observation, possibly missing insights on user experience. For instance, users did not report the lack of navigation cues (e.g., arrow icons) for swiping between lesion images nor missing instructions for using the chatbot. Additionally, some images failed to load fully, likely due to poor internet connectivity.

Additional deviations from the study protocol are given in supporting information file S2 Table.

### Implications for app development

Based on user feedback, several improvements are recommended to enhance app usability and effectiveness:

- **Offline functionality**: Offline access to ensure usability in areas with poor internet connectivity.
- **Localized content**: Translation of the app into local languages and the inclusion of images with darker skin tones to improve diagnostic accuracy and relevance.
- **Enhanced interactivity**: The addition of such features as push notifications, reminders, and gamification to improve user engagement and encourage consistent use.
- **Surveillance features**: The addition of surveillance features, such as data-saving functionality, to enable the recording of patient data and support for disease monitoring.

### Policy implications

- **Ensuring accessibility for widespread use:** The WHO should continue to provide the app free of charge to prevent out-of-pocket costs limiting widespread use.
- **Enhancing user engagement:** To increase user engagement, the WHO should collaborate with developers to integrate reminders and just-in-time notifications.
- **Incorporating the app into healthcare worker training:** training modules about the app should be integrated into healthcare worker training curriculum, and briefing sessions routinely organized by health system programs, that cover both dermatological/parasitological fundamentals and app usage. Quick-reference guides and video tutorials should be developed to support user onboarding.
- **Disseminating the WHO SkinNTDs App:** Improvements to the app should be followed by targeted efforts to raise awareness among users. A marketing strategy highlighting app benefits (e.g., free access, diagnostic support) is essential. mHealth apps can be promoted through both paid and unpaid strategies across various communication channels, and the impact of these strategies is measurable by download numbers and user engagement [30]. Our findings suggest promotional messages should emphasize the reputable source and free accessibility.

### Recommendations for improving app quality

Recommendations for improving the quality of the SkinNTDs app are summarized in Table 5, classified under uMARS dimensions.

**Table 5:** Synthesis of recommendations for improving app quality under uMARS dimensions.

Num	Recommendations	Recommendation target
	**Dimension: *Functionality***	
1.	Ensure intuitive navigation and logical screen flow.	App Developers/UX Designers
2.	Add offline functionality for areas with poor internet access.	App Developers/UX Designers
3.	Provide training to healthcare workers on how to use the app effectively.	Digital Health Implementers
4.	Develop user guides and FAQs to assist new users.	Digital Health Implementers
5.	Advocate for internet connectivity improvements in rural areas to support app functionality.	Ministry of Public Health/WHO
6.	Fund technical support for app maintenance and updates.	Ministry of Public Health/WHO
7.	**Dimension: *Aesthetics***	
8.	Improve visual design (e.g., high-quality graphics, consistent styling).	App Developers/UX Designers
9.	Ensure the app is accessible to users with disabilities (e.g., colorblind-friendly design).	Digital Health Implementers
10.	Provide funding for UX improvements based on user feedback.	Ministry of Public Health/WHO
	**Dimension: *Engagement***	
11.	Incorporate gamification (e.g., quizzes) and social features (e.g., sharing progress, peer support), and just-in-time push notifications to boost engagement and encourage consistent use.	App Developers/UX Designers
12.	Add a data-saving option to assist case notification and surveillance	App Developers/UX Designers
13.	Personalize content based on user preferences and usage patterns.	App Developers/UX Designers
14.	Organize workshops to demonstrate app features and benefits.	Digital Health Implementers
15.	Encourage peer-to-peer sharing of the app within healthcare networks.	Digital Health Implementers
16.	Monitor engagement metrics (e.g., frequency of use) to identify drop-off points.	Digital Health Implementers
17.	Launch awareness campaigns to promote the app among healthcare workers and the public.	Ministry of Public Health/WHO
18.	Develop partnerships with local organizations to increase app adoption.	Ministry of Public Health/WHO
19.	Provide incentives for healthcare workers to use the app (e.g., certifications, recognition).	Ministry of Public Health/WHO
	**Dimension: *Information Quality***	
20.	Ensure all medical information is accurate, up-to-date, and evidence-based.	App Developers/UX Designers
21.	Include visual aids (e.g., images, graphs) to enhance understanding.	App Developers/UX Designers
22.	Train healthcare workers on how to interpret and use the app’s information effectively.	Digital Health Implementers
23.	Update country data and add disaggregation to regional or district level statistics.	Digital Health Implementers
24.	Collaborate with dermatologists and other organizations to regularly validate and update app content.	Ministry of Public Health/WHO
25.	Fund the development of additional educational materials (e.g., videos, infographics, user guide).	Ministry of Public Health/WHO
	**Dimension: *Subjective Quality***	
26.	Address user complaints and suggestions in app updates.	App Developers/UX Designers
27.	Provide ongoing support to users through help desks, WhatsApp groups or chatbots.	Digital Health Implementers
28.	Publish reports on app performance and user satisfaction to build trust and credibility.	Ministry of Public Health/WHO
	**Dimension: *Perceived Impact***	
29.	Add features to track user progress and outcomes (e.g., diagnostic accuracy, patient cases).	App Developers/UX Designers
30.	Collect and showcase testimonials or case studies from satisfied users.	App Developers/UX Designers
31.	Conduct impact evaluations to measure app effectiveness in improving health outcomes.	Digital Health Implementers
32.	Share success stories and best practices with stakeholders.	Digital Health Implementers
33.	Use impact data to advocate for scaling up the app to other regions or countries.	Ministry of Public Health/WHO
34.	Fund research to assess the long-term impact on skin NTDs management.	Ministry of Public Health/WHO

### Future research directions

Future research should focus on:

- **Expanding the scope of the app**: Including other common skin conditions and integrating the app with broader health initiatives, such as HIV co-morbidity management, could enhance its utility.
- **Context-specific evaluations**: Conducting evaluations in LMICs and other regions including Americas, Asia and Europe to validate these findings and identify additional context-specific challenges. Indeed, although this study is focused in Cameroon, the diseases embedded in the app are not only from west Africa context and they are endemic from all the tropical area worldwide. Moreover, in the global world we live, people travel at any point of the planet and diseases may travel with the people. For example, a recent review highlighted rising of cutaneous leishmaniasis (CL) and other skin diseases cases observed in refugee settlements, migrant populations and travelers, particularly in Lebanon, Jordan, Europe and North America [31]. Many healthcare providers in the U.S. and Europe lack experience diagnosing and managing CL, leading to misdiagnosis or treatment delays [32]. Thus, dissemination efforts towards wide use of the app and context-specific evaluations should also target those regions where skin NTDs and common skin diseases covered by the app are becoming prevalent.
- **Exploring the role of community health volunteers in app integration:** Community health volunteers are an integral part of the health system, working closely with populations in remote areas. Future studies should assess the acceptability and user experience of the WHO SkinNTDs app among this group. This would support integration of the app within the health system, as community health volunteers are often involved in multiple health programs.
- **Longitudinal studies**: The long-term impact of the app on clinical outcomes and its usability among community health workers, who play a critical role in NTD case identification, should be assessed. These workers can refer patients in early stage of disease [33].
- **Further research on app effectiveness in skin NTD case-finding:** Determining the extent to which the app contributes to skin NTD case-finding is crucial. Future research should assess its effectiveness in real-world case-finding and surveillance, providing an opportunity to update country statistics within the ‘Global Index’ function.

## Conclusions

The WHO SkinNTDs app demonstrated moderate usability and acceptability among healthcare workers in Cameroon, with an overall quality mean score of 3.61/5. Perceived impact was the highest-rated subdomain (3.88/5), and engagement the lowest-rated one (3.50/5), indicating a need for improved interactivity. Training to use the app, and support for surveillance features and internet-dependent functions were significantly associated with higher app quality scores. Participants suggested key improvements, including better translations, enhanced image quality, and the addition of more conditions.

For app developers, these findings highlight the importance of user-centered design [34] and context-specific adaptations. Prioritizing offline functionality and localized content will contribute to address challenges in resource-limited settings, such as unreliable internet and linguistic diversity. Enhancing interactivity and customization features can improve user engagement, while expanding content to include more conditions will increase utility.

For policymakers, the study underscores the critical role of training in both app use and dermatology to support effective app adoption. Incorporating app training into structured curricula or routine briefing sessions for healthcare workers can enhance the app’s possible contribution as a capacity-building tool. Furthermore, integrating the app into existing health system programs, especially those focused on disease surveillance and community health, could strengthen NTD management in underserved areas.

For future research, evaluations in other regions and LMICs are needed to validate these findings and identify additional context-specific challenges. Longitudinal studies should assess the long-term impact on clinical outcomes, while research on usability among community health workers can further enhance its reach in rural settings.

In summary, the WHO SkinNTDs app demonstrates potential as a capacity-building tool to improve skin NTD diagnosis and management in resource-limited settings. By addressing user feedback, enhancing offline functionality, and providing targeted training, the app can become a more effective tool. Providing actionable insights for developers and policymakers to optimize digital health interventions for NTDs, this study emphasizes the importance of context-specific evaluations and user-centered design. Developers should prioritize offline functionality, localized content, and enhanced interactivity to improve user engagement and adoption. Future research should focus on longitudinal studies to assess the long-term impact of the app on case-finding and clinical outcomes and explore its usability among community health workers.

## Supporting information

S1 Appendix Guide for focus group discussions. (DOCX)

S2 Appendix Definitions of uMARS variables and strategic-level key informants’ questionnaire. (DOCX)

S3 Appendix Comparison between uMARS app quality mean score and the main demographic variables of participants. (DOCX)

S4 Appendix Multiple linear regression. (DOCX)

S5 Appendix Results of qualitative data analysis. (DOCX)

S1 Table Details of participants in focus group sessions. (DOCX)

S2 Table Deviations from the study protocol. (DOCX)

S1 Dataset Data files (compressed). (ZIP)
