# Supplementary material for: Usability and quality evaluation of the World Health Organization SkinNTDs app among frontline health workers in Cameroon: A mixed methods study
Source: PLoS Negl Trop Dis. 2025 Sep 10;19(9):e0013461. doi: 10.1371/journal.pntd.0013461 (PMC12422481; doi:10.1371/journal.pntd.0013461)
Supplement: S1 Appendix — (DOCX) [file pntd.0013461.s001.docx]

**Supporting information file.**

**S1 Appendix. Guide for focus group discussions.**

**Title of the study:** Assessing the Quality of the World Health Organization SkinNTDs App in Cameroon: A Cross-sectional Study

**Target participants:** Healthcare workers providing services in public or private health settings.

**Targeted number of participants:** 10–15.

**Venue:** TBA.

**Materials:** Notebook, pen, and mobile phone (for audio recording) for research team.

**Informed consent for participants:** Obtain informed consent from participants. Inform participants that attending the session indicates de facto consent.

**Consent of session recording:** Inform participants that written notes will be taken by researchers and anonymized. Explain that the session will be audio-recorded on mobile phone.

**Confidentiality and data protection** Inform participants that the audio record will not be shared with anyone outside the research team.

**Duration:** 60–120 min

**Outline for the focus group discussion:**

Brief presentation of the interviewer and the study

1. Have you ever used a mobile health app? If yes, which one?
2. Have you used the SkinNTDs app? For how long? In what situations?
3. What is your overall opinion of the SkinNTDs App?
4. What are the features that you have identified in the app?
5. What features do you think are essential to the app? That is, the features you would not change. Why?
6. What features do you think are the least useful? That is, the features you think could be removed. Why?
7. What features must the app have for you to use it or recommend it to someone else?
8. Do you see the app being incorporated as a standard medical device in your workplace? Why?
9. Having used the app, who do you think is the ideal final user?
10. What do you think would be the best option for disseminating the App? How would you ensure the maximum number of ideal final users become aware of the app and download it?
11. Do you think that 5 days were enough time to test the App and be able to answer the MARS questionnaire?
12. Are there any challenges that you foresee in the dissemination of the app, its installation and use?
13. How do you think these challenges could be addressed?
14. Would you like to add anything else, or talk about another aspect that you feel is relevant to the app?
