# Supplementary material for: Usability and quality evaluation of the World Health Organization SkinNTDs app among frontline health workers in Cameroon: A mixed methods study
Source: PLoS Negl Trop Dis. 2025 Sep 10;19(9):e0013461. doi: 10.1371/journal.pntd.0013461 (PMC12422481; doi:10.1371/journal.pntd.0013461)
Supplement: S2 Appendix — (DOCX) [file pntd.0013461.s002.docx]

**Supporting information file.**

**S2 Appendix.** **Questionnaire for key strategic-level informants on the usability of the WHO SkinNTDs app and Definitions of uMARS subdomains and associated variables.**

**PART 1**: Definitions of uMARS subdomains and associated variables.

**Table 1.** **Definitions of uMARS subdomains and associated variables.**

| **Subdomains** | | **Definitions** |
| --- | --- | --- |
| ***Engagement* score** | | ***Measures how engaging and enjoyable the app is for users.*** |
|  | Entertainment | How fun or entertaining the app is. |
|  | Interest | How interesting the app is; strategies to increase engagement. |
|  | Customization | Availability of settings/preferences (e.g., sound, notifications). |
|  | Interactivity | Allows user input, feedback, prompts (e.g., reminders, sharing). |
|  | Target group | Appropriateness of content (language, design) for the target audience. |
| ***Functionality* score** | | ***Measures how well the app functions technically.*** |
|  | Performance | Accuracy and speed of app features and components (e.g., buttons, menus). |
|  | Ease of use | How easy it is to learn and use the app; clarity of menu labels/icons. |
|  | Navigation | Logical and accurate movement between screens; presence of necessary links. |
|  | Gestural design | Consistency and intuitiveness of interactions (e.g., taps, swipes). |
| ***Aesthetic* score** | | ***Measures the visual appeal and design quality of the app.*** |
|  | Layout | Arrangement and size of buttons/icons/menus/content on the screen. |
|  | Graphics | Quality/resolution of graphics used in the app. |
|  | Visual appeal | Overall visual attractiveness of the app. |
| ***Information* score** | | ***Measures the quality, accuracy, and relevance of app content.*** |
|  | Quality of information | Whether the app contains what is described in the app store. |
|  | Quantity of information | Whether the app has specific, achievable goals. |
|  | Visual information | Correctness, relevance, and coherence of app content. |
|  | Credibility of source | Extent of coverage within scope of the app; comprehensiveness and conciseness. |
| ***Subjective items score*** | | ***Measures user satisfaction and willingness to recommend the app.*** |
|  | Would you recommend the app? | Likelihood of recommending the app to others. |
|  | How many times do you use the app? | Expected frequency of app use in the next 12 months. |
|  | Would you pay for the app? | Whether users would pay for the app. |
|  | Overall (star) rating | Overall star rating of the app. |
| ***Perceived impact score*** | | ***Measures the perceived impact of app on knowledge and diagnostic efficiency.*** |
|  | The app increases knowledge about sNTDs | Likelihood of the app increasing knowledge about skin NTDs. |
|  | The app will help for sNTDs diagnosis | Likelihood of the app helping users diagnose skin NTDs faster and more efficiently. |

**PART 2**: Questionnaire for key strategic-level informants on the usability of the WHO SkinNTDs app

**Features of the WHO SkinNTDs app**

1. What features should definitely be included in the app for you to recommend it to others? Why?
2. Which features do you think are the least essential and could be removed? Why?
3. What modifications would you propose to improve the app?

**Use Cases for the WHO SkinNTDs application**

1. After exploring the app, who do you think would be the ideal end user?
2. When would be the best time for this ideal user to use the app, or in what cases/situations do you think the app should be used in the field by the ideal user?
3. Do you foresee the app being integrated as a standard medical device in your service/program? Why?
4. What would be the best way to disseminate or promote the app?
5. When would be the best time to promote the app within the NTD (Neglected Tropical Diseases) control program?

**Specific perspectives at the strategic level of the NTD program**

1. As a strategic-level resource person in the NTD program, what prerequisites would you indicate before adopting this app among the tools used for the detection/surveillance of skin-related NTDs?
2. Field health workers have suggested that the app be translated into the following foreign languages (Spanish, German, Chinese, English, Arabic, French, Portuguese, Russian) and local languages (Fufulde, Toupouri, Massa, Mafa, Mousgoum, Moundang, Hausa, Ewondo, Guiziga, Baya, Laka). What is your opinion?
3. Are there are any aspects we have not addressed that you would like to discuss with the research team? If so, what are they?
