# Supplementary material for: Usability and quality evaluation of the World Health Organization SkinNTDs app among frontline health workers in Cameroon: A mixed methods study
Source: PLoS Negl Trop Dis. 2025 Sep 10;19(9):e0013461. doi: 10.1371/journal.pntd.0013461 (PMC12422481; doi:10.1371/journal.pntd.0013461)
Supplement: S3 Appendix — (DOCX) [file pntd.0013461.s003.docx]

**Supporting information file. S3 Appendix.**

**Analysis 1. Comparison between uMARS app quality mean score versus the main demographic variables of participants in the study.**

| **Variables compared and statistical tests performed** | |  | **Value** | | **Degree of freedom** | | **P value** | **Effect**  **size** | **Mean difference** | **95% mean difference Confidence Interval** | |
| --- | --- | --- | --- | --- | --- | --- | --- | --- | --- | --- | --- |
|  | |  |  | |  | |  |  |  | Lower | Upper |
| **App quality mean score vs age** | |  | |  | |  | |  |  |  |  |
|  | Levene statistic | 0.680 | | 179 | | 0.508 | |  |  |  |  |
|  | Shapiro wilk test | 0.985 | |  | | 0.050 | |  |  |  |  |
|  | ANOVA | 0.181 | | 179 | | 0.835 | | 0.002^d^ |  |  |  |
| **App quality mean score vs sex** | |  | |  | |  | |  |  |  |  |
|  | Levene statistic | 1.720 | | 179 | | 0.191 | |  |  |  |  |
|  | Shapiro wilk test | 0.985 | |  | | 0.048**^a^** | |  |  |  |  |
|  | Mann-Whitney U | 3584 | |  | | 0.892 | | 0.012^e^ | -0.017 | -0.237 | 0.221 |
| **App quality mean score vs** **type of survey participant** | |  |  |  |  | |  |  |  |  |  |
|  | Levene statistic | 0.694 | | 179 | | 0.406 | |  |  |  |  |
|  | Shapiro wilk test | 0.986 | |  | | 0.064 | |  |  |  |  |
|  | Student's t | 1.330 | | 178 | | 0.186 | | 0.200^f^ | 0.145 | -0.071 | 0.361 |
| **App quality mean score vs** **interested for a desktop version of the app** | | | |  |  | |  |  |  |  |  |
|  | Levene statistic | 15.005 | | 179 | | <0.001**^b^** | |  |  |  |  |
|  | Shapiro wilk test | 0.990 | |  | | 0.235 | |  |  |  |  |
|  | Welch's t | -1.080 | | 17.3 | | 0.295 | | 0.322^f^ | 0.296 | -0.282 | 0.873 |
| **App quality mean score vs trained to use the app** | |  |  |  |  | |  |  |  |  |  |
|  | Levene statistic | 2.860 | | 179 | | 0.093 | |  |  |  |  |
|  | Shapiro wilk test | 0.989 | |  | | 0.180 | |  |  |  |  |
|  | Student's t | 2.570 | | 178 | | **0.005^c^** | | **0.405^f^** | **0.291** | **0.104** | **Inf** |
| **App quality mean score vs** **work context** | |  |  |  |  | |  |  |  |  |  |
|  | Levene statistic | 1.910 | | 179 | | 0.152 | |  |  |  |  |
|  | Shapiro wilk test | 0.983 | |  | | 0.032**^a^** | |  |  |  |  |
|  | Kruskal-Wallis test | 0.273 | | 2 | | 0.872 | | 0.001^g^ |  |  |  |
| **App quality mean score vs region** | |  |  |  |  | |  |  |  |  |  |
|  | Levene statistic | 1.640 | | 133 | | 0.167 | |  |  |  |  |
|  | Shapiro wilk test | 0.979 | |  | | 0.041**^a^** | |  |  |  |  |
|  | Kruskal-Wallis test | 0.763 | | 4 | | 0.943 | | 0.006^g^ |  |  |  |
| **App quality mean score vs** **frequency of dealing with patients with skin diseases** | | | |  |  | |  |  |  |  |  |
|  | Levene statistic | 0.466 | | 179 | | 0.706 | |  |  |  |  |
|  | Shapiro wilk test | 0.986 | |  | | 0.061 | |  |  |  |  |
|  | ANOVA | 1.360 | | 179 | | 0.258 | | 0.023^d^ |  |  |  |
| **App quality mean score vs** **dermatology experience** | |  |  |  |  | |  |  |  |  |  |
|  | Levene statistic | 0.883 | | 179 | | 0.415 | |  |  |  |  |
|  | Shapiro wilk test | 0.985 | |  | | 0.046**^a^** | |  |  |  |  |
|  | Kruskal-Wallis test | 2.070 | | 2 | | 0.355 | | 0.012^g^ |  |  |  |
| **App quality mean score vs** **how participants heard about the app** | | |  |  |  | |  |  |  |  |  |
|  | Levene statistic | 0.196 | | 178 | | 0.899 | |  |  |  |  |
|  | Shapiro wilk test | 0.985 | |  | | 0.058 | |  |  |  |  |
|  | ANOVA | 0.328 | | 178 | | 0.805 | | 0.006^d^ |  |  |  |
| **App quality mean score vs willingness to pay for the app** | |  |  |  |  | |  |  |  |  |  |
|  | Levene statistic | 0.779 | | 179 | | 0.460 | |  |  |  |  |
|  | Shapiro wilk test | 0.988 | |  | | 0.124 | |  |  |  |  |
|  | ANOVA | 2.940 | | 179 | | 0.056 | | 0.032^d^ |  |  |  |
| **App quality mean score vs** **patients records** | |  |  |  |  | |  |  |  |  |  |
|  | Levene statistic | 10.700 | | 179 | | 0.001**^b^** | |  |  |  |  |
|  | Shapiro wilk test | 0.988 | |  | | 0.119 | |  |  |  |  |
|  | Welch's t | 0.376 | | 10.5 | | 0.715 | | 0.140^f^ | 0.135 | -0.663 | 0.934 |
| **App quality mean score vs add surveillance features to the app** | |  |  |  |  | |  |  |  |  |  |
|  | Levene statistic | 2.75 | | 178 | | 0.099^b^ | |  |  |  |  |
|  | Shapiro wilk test | 0.993 | |  | | 0.506 | |  |  |  |  |
|  | Welch's t | 3.360 | | 178 | | **0.017^c^** | | **0.787^f^** | **0.686** | **0.172** | **Inf** |
| **App quality mean score vs** **translate the app** | |  |  |  |  | |  |  |  |  |  |
|  | Levene statistic | 1.680 | | 179 | | 0.196 | |  |  |  |  |
|  | Shapiro wilk test | 0.985 | |  | | 0.051 | |  |  |  |  |
|  | Student's t | 0.247 | | 178 | | 0.805 | | 0.038 | 0.027 | -0.192 | 0.247 |
| **App quality mean score vs adding internet-depending functions to the app** | | | |  |  | |  |  |  |  |  |
|  | Levene statistic | 2.820 | | 179 | | 0.026^b^ | |  |  |  |  |
|  | Shapiro wilk test | 0.991 | |  | | 0.345 | |  |  |  |  |
|  | Kruskal-Wallis test | 19 | | 4 | | **<0.001^c^** | | **0.106^g^** |  |  |  |

^a^ normality assumption violated; ^b^ variance homogeneity assumption violated; ^c^ statistical significance

^d^ η² ; ^e^ Rank biserial correlation ; ^f^ Cohen’s d ; ^g^ ε^2^

**Reference table. Interpreting effect size values** *(Source: Maher JM, Markey JC, Ebert-May D. The Other Half of the Story: Effect Size Analysis in Quantitative Research. CBE—Life Sci Educ. 2013;12: 345–351. doi:10.1187/cbe.13-04-0082)*

| **Effect size measure** | **Small**  **effect size** | **Medium**  **effect size** | **Large**  **effect size** | **Very large**  **effect size** |
| --- | --- | --- | --- | --- |
| **Cohen’s d (or one of its variants)** | 0.20 | 0.50 | 0.80 | 1.30 |
| **Pearson’s r** | 0.10 | 0.30 | 0.50 | 0.70 |
| **Eta-squared** | 0.01 | 0.06 | 0.14 |  |

**Analysis 2. Independent samples T-Test : uMARS scores in Professionals vs Students**

|  |  |  |  |  |  | **95% Confidence Interval** | |  |
| --- | --- | --- | --- | --- | --- | --- | --- | --- |
|  | **Statistic**  **(Welch's t)** | **df** | **p** | **Mean difference** | **SE difference** | **Lower** | **Upper** | **Effect Size**  (Cohen's d) |
| **App quality mean score** | 1.332 | 170 | 0.185 | 0.145 | 0.109 | -0.070 | 0.361 | 0.200 |
| **Engagement mean score** | 1.457 | 171 | 0.147 | 0.180 | 0.124 | -0.064 | 0.425 | 0.218 |
| **Functionality mean score** | 1.362 | 169 | 0.175 | 0.187 | 0.138 | -0.084 | 0.459 | 0.204 |
| **Aesthetics mean score** | 0.423 | 173 | 0.673 | 0.055 | 0.129 | -0.200 | 0.309 | 0.063 |
| **Information mean score** | 1.347 | 164 | 0.180 | 0.159 | 0.118 | -0.074 | 0.391 | 0.203 |
| **App subjective mean score** | **2.241** | **145** | **0.013** | **0.222** | **0.099** | **0.058** | **Inf** | **0.341** |
| **App perceived impact mean score** | **8.157** | **178** | **<.001** | **0.833** | **0.102** | **0.664** | **Inf** | **1.210** |

 Note. Hₐ μ_Professional_ ≠ μ_Student ;_ Hₐ μ_Professional_ > μ_Student_if statistical difference

**Assumptions**

| Normality Test (Shapiro-Wilk) | | |
| --- | --- | --- |
|  | **W** | **p** |
| **App quality mean score** | 0.986 | 0.064 |
| **Engagement mean score** | 0.981 | 0.015 |
| **Functionality mean score** | 0.957 | <.001 |
| **Aesthetics mean score** | 0.974 | 0.002 |
| **Information mean score** | 0.960 | <.001 |
| **App subjective mean score** | 0.965 | <.001 |
| **App perceived impact mean score** | 0.902 | <.001 |
| Note. A low p-value suggests a violation of the assumption of normality | | |

| Homogeneity of Variances Test (Levene's) | | | | |
| --- | --- | --- | --- | --- |
|  | **F** | **df** | **df2** | **p** |
| **App quality mean score** | 0.694 | 1 | 178 | 0.406 |
| **Engagement mean score** | 0.011 | 1 | 178 | 0.918 |
| **Functionality mean score** | 0.086 | 1 | 178 | 0.769 |
| **Aesthetics mean score** | 0.894 | 1 | 178 | 0.346 |
| **Information mean score** | 2.27e-4 | 1 | 178 | 0.988 |
| **App subjective mean score** | 4.605 | 1 | 178 | 0.033 |
| **App perceived impact mean score** | 0.535 | 1 | 178 | 0.465 |
| Note. A low p-value suggests a violation of the assumption of equal variances | | | | |

| Group Descriptives | | | | | | |  |  |
| --- | --- | --- | --- | --- | --- | --- | --- | --- |
|  | **Group** | **N** | **Mean** | **Median** | **SD** | **SE** | |  |
| **App quality mean score** | **Professional** | 101 | 3.68 | 3.74 | 0.736 | | 0.073 | |
|  | **Student** | 79 | 3.53 | 3.57 | 0.718 | | 0.081 | |
| **Engagement mean score** | **Professional** | 101 | 3.57 | 3.60 | 0.848 | | 0.084 | |
|  | **Student** | 79 | 3.39 | 3.40 | 0.806 | | 0.091 | |
| **Functionality mean score** | **Professional** | 101 | 3.77 | 4.00 | 0.926 | | 0.092 | |
|  | **Student** | 79 | 3.58 | 3.75 | 0.910 | | 0.102 | |
| **Aesthetics mean score** | **Professional** | 101 | 3.61 | 3.67 | 0.897 | | 0.089 | |
|  | **Student** | 79 | 3.55 | 3.67 | 0.825 | | 0.093 | |
| **Information mean score** | **Professional** | 101 | 3.76 | 3.83 | 0.763 | | 0.076 | |
|  | **Student** | 79 | 3.60 | 3.67 | 0.801 | | 0.090 | |
| **App subjective mean score** | **Professional** | 101 | 3.41 | 3.50 | 0.569 | | 0.057 | |
|  | **Student** | 79 | 3.19 | 3.25 | 0.721 | | 0.081 | |
| **App perceived impact mean score** | **Professional** | 101 | 4.24 | 4.00 | 0.754 | | 0.075 | |
|  | **Student** | 79 | 3.41 | 3.00 | 0.615 | | 0.069 | |

Professionals (n=101) scored the app's perceived impact significantly higher than students (n=79) (mean = 4.2 vs 3.4, mean difference =0.833, 95%CI [0.102; Inf[, p<0.001, Cohen’s d = 1.210), and app’s subjective mean score than students (mean = 3.4 vs 3.2, mean difference =0.833, 95%CI [0.664; Inf[, p<0.001, Coehn’s d = 0.341). The tests showed no significant differences in quality, engagement, functionality, aesthetics or information mean scores (all p>0.15).

**Analysis 3. Independent samples T-Test : Perceived impact and subjective perception in Professionals vs Students**

|  |  |  |  |  |  | **95% Confidence Interval** | |  |
| --- | --- | --- | --- | --- | --- | --- | --- | --- |
|  | **Statistic**  **(Welch's t)** | **df** | **p** | **Mean difference** | **SE difference** | **Lower** | **Upper** | **Effect Size**  (Cohen's d) |
| **Would you recommend this app to people who might benefit from it?** | 0.422 | 169.0 | 0.673 | 0.079 | 0.186 | -0.289 | 0.447 | 0.063 |
| **How many times do you think you would use this app in the next 12 months?** | 1.496 | 168.3 | 0.136 | 0.231 | 0.154 | -0.074 | 0.535 | 0.225 |
| **Would you pay for this app?** | 1.169 | 165.2 | 0.244 | 0.134 | 0.115 | -0.093 | 0.361 | 0.176 |
| **What is your overall star rating of the app?** | **3.939** | **83.1** | **<.001** | **0.443** | **0.112** | **0.256** | **Inf** | **0.624** |
| **This app is likely to increase my knowledge about skin NTDs (Perceived impact 2)** | 1.077 | 171.4 | 0.283 | 0.131 | 0.122 | -0.109 | 0.371 | 0.161 |
| **This app is likely to help me come up faster and more efficiently with a diagnosis on skin NTD** | **8.723** | **129.9** | **<.001** | **1.534** | **0.176** | **1.243** | **Inf** | **1.340** |

 Note. Hₐ μ_Professional_ ≠ μ_Student ;_ Hₐ μ_Professional_ > μ_Student_if statistical difference

**Assumptions**

| **Normality Test (Shapiro-Wilk)** | | |
| --- | --- | --- |
|  | **W** | **p** |
| **Would you recommend this app to people who might benefit from it?** | 0.808 | <.001 |
| **How many times do you think you would use this app in the next 12 months?** | 0.928 | <.001 |
| **Would you pay for this app?** | 0.843 | <.001 |
| **What is your overall star rating of the app?** | 0.795 | <.001 |
| **This app is likely to increase my knowledge about skin NTDs (Perceived impact 2)** | 0.819 | <.001 |
| **This app is likely to help me come up faster and more efficiently with a diagnosis on skin NTD** | 0.880 | <.001 |
| Note. A low p-value suggests a violation of the assumption of normality | | |

| **Homogeneity of Variances Test (Levene's)** | | | | |
| --- | --- | --- | --- | --- |
|  | **F** | **df** | **df2** | **p** |
| **Would you recommend this app to people who might benefit from it?** | 0.138 | 1 | 178 | 0.710 |
| **How many times do you think you would use this app in the next 12 months?** | 0.041 | 1 | 178 | 0.839 |
| **Would you pay for this app?** | 1.320 | 1 | 178 | 0.252 |
| **What is your overall star rating of the app?** | 165.388 | 1 | 178 | <.001 |
| **This app is likely to increase my knowledge about skin NTDs (Perceived impact 2)** | 0.1983 | 1 | 178 | 0.657 |
| **This app is likely to help me come up faster and more efficiently with a diagnosis on skin NTD** | 46.815 | 1 | 178 | <.001 |
| Note. A low p-value suggests a violation of the assumption of equal variances | | | | |

**Reference table. Interpreting effect size values** *(Source: Maher JM, Markey JC, Ebert-May D. The Other Half of the Story: Effect Size Analysis in Quantitative Research. CBE—Life Sci Educ. 2013;12: 345–351. doi:10.1187/cbe.13-04-0082)*

| **Effect size measure** | **Small**  **effect size** | **Medium**  **effect size** | **Large**  **effect size** | **Very large**  **effect size** |
| --- | --- | --- | --- | --- |
| **Cohen’s d (or one of its variants)** | 0.20 | 0.50 | 0.80 | 1.30 |
| **Pearson’s r** | 0.10 | 0.30 | 0.50 | 0.70 |
| **Eta-squared** | 0.01 | 0.06 | 0.14 |  |

| Group Descriptives | | | | | | |
| --- | --- | --- | --- | --- | --- | --- |
|  | **Group** | **N** | **Mean** | **Median** | **SD** | **SE** |
| **App Subjective sub-domain** | | | | | | |
| **Would you recommend this app to people who might benefit from it?** | **Professional** | 101 | 3.99 | 5.00 | 1.253 | 0.125 |
|  | **Student** | 79 | 3.91 | 4.00 | 1.232 | 0.139 |
| **How many times do you think you would use this app in the next 12 months?** | **Professional** | 101 | 3.76 | 4.00 | 1.031 | 0.103 |
|  | **Student** | 79 | 3.53 | 4.00 | 1.023 | 0.115 |
| **Would you pay for this app?** | **Professional** | 101 | 1.88 | 2.00 | 0.752 | 0.075 |
|  | **Student** | 79 | 1.75 | 2.00 | 0.776 | 0.087 |
| **What is your overall star rating of the app?** | **Professional** | 101 | 4.00 | 4.00 | 0.200 | 0.020 |
|  | **Student** | 79 | 3.56 | 3.00 | 0.984 | 0.111 |
| **App Perceived impact sub-domain** | | | | | | |
| **This app is likely to increase my knowledge about skin NTDs (Perceived impact 2)** | **Professional** | 101 | 4.26 | 4.00 | 0.833 | 0.083 |
|  | **Student** | 79 | 4.13 | 4.00 | 0.790 | 0.089 |
| **This app is likely to help me come up faster and more efficiently with a diagnosis on skin NTD** | **Professional** | 101 | 4.22 | 4.00 | 0.901 | 0.089 |
|  | **Student** | 79 | 2.68 | 3.00 | 1.345 | 0.151 |

Professionals’ high rating in app's subjective mean score and app’s perceived impact score were mainly driven by their overall star rating of the app (mean = 4.0 vs 3.6, mean difference =0.443, 95%CI [0.256; Inf[, p<0.001, Cohen’s d = 0.624) and their perception that the app is likely to help as diagnostic support tool (mean = 4.2 vs 2.7, mean difference =1.543, 95%CI [1.243; Inf[, p<0.001, Cohen’s d = 1.340).

**Analysis 4. Cross Table: differences in other survey variables in Professionals vs Students**

|  | **N** | **Professional** | **Student** | **Test Statistic (Pearson’s**  **Chi-squared test)** |
| --- | --- | --- | --- | --- |
|  |  | **(N=101)** | **(N=79)** |  |
| Have you been trained to use the App? : No | 180 | 0.8   76/101 | 0.5  43/79 | χ **^2^(df=1)=8.57, P<0.01** |
| What is your knowledge about Mobile technologies | 180 |  |  | χ **^2^(df=2)**=0.66, P=0.72 |
| High |  | 0.3   26/101 | 0.2  18/79 |  |
| Medium |  | 0.7   72/101 | 0.7  57/79 |  |
| Low |  | 0.0   3/101 | 0.1   4/79 |  |
| Do you have experience in dermatology? | 180 |  |  | χ **^2^(df=2)**=3.92, P=0.14 |
| Not trained & not experienced |  | 0.4   40/101 | 0.5  43/79 |  |
| Not trained but experienced |  | 0.5   54/101 | 0.4  32/79 |  |
| Trained or experienced |  | 0.1   7/101 | 0.1   4/79 |  |
| Trained on Skin NTDs diagnostic & management : No | 178 | 0.7  70/99 | 0.9  68/79 | χ **^2^(df=1)=5.96, P=0.01** |
| Interested for desktop version : No | 180 | 0.0   5/101 | 0.2  12/79 | χ **^2^(df=1)=5.43, P=0.02** |
| Add data saving section : No | 180 | 0.0   3/101 | 0.1   8/79 | χ **^2^(df=1)**=3.96, P=0.05 |
| Add sNTDs surveillance features : No | 180 | 0.0   4/101 | 0.1   9/79 | χ **^2^1(df=1)**==3.65, P=0.06 |
| Perception about adding internet-dependent functions | 180 |  |  | χ**^2^(df=4)**=7.57, P=0.11 |
| I mind a lot. I hardly never have internet connection on my phone |  | 0.1   8/101 | 0.2  17/79 |  |
| I mind it. Most of the time I do not have access to the internet on my phone |  | 0.3   30/101 | 0.3  23/79 |  |
| I do not care. Sometimes I have internet on my phone |  | 0.1   7/101 | 0.0   3/79 |  |
| I am up for it. Most of the time I have internet on my phone |  | 0.5   46/101 | 0.4  29/79 |  |
| I think it would be great. I always have internet on my phone |  | 0.1   10/101 | 0.1   7/79 |  |

 N is the number of non-missing value. No is reference response vs Yes.
