# Supplementary material for: Usability and quality evaluation of the World Health Organization SkinNTDs app among frontline health workers in Cameroon: A mixed methods study
Source: PLoS Negl Trop Dis. 2025 Sep 10;19(9):e0013461. doi: 10.1371/journal.pntd.0013461 (PMC12422481; doi:10.1371/journal.pntd.0013461)
Supplement: S4 Appendix — (DOCX) [file pntd.0013461.s004.docx]

**Supporting information file.**

**S3 Appendix.** **Multiple linear regression.**

**Purpose of the initial analysis – model 1**

The regression analysis examines the statistical association between the app quality mean score and three independent variables:

1. **Participant has undergone training to use the app**
2. **Participant in favor of the addition of surveillance features**
3. **Participant in favor of the addition of internet-dependent functions**

These variables are those initially found statistically associated with the quality mean score. In this, we aimed to determine whether these factors altogether significantly influence user perception of app quality.

| **Table 1. Model Fit Measures** | | | | | | | |
| --- | --- | --- | --- | --- | --- | --- | --- |
|  | | | | **Overall Model Test** | | | |
| **Model** | **R²** | **Adjusted R²** | **AIC** | **F** | **df1** | **df2** | **p** |
| 1 | 0.231 | 0.204 | 365 | 8.64 | 6 | 173 | <.001 |
| Note. Models estimated using sample size of N=180 | | | | | | | |

**Model fit and significance**

- The model explains **23.1%** of the variance in app quality scores (**R² = 0.231, adjusted R² = 0.204**), indicating a low explanatory power.
- The **F-test** is statistically significant (**F(6,173) = 8.64, p < 0.001**), suggesting that at least one of the predictor variables contributes significantly to the model.

| **Table 2. Omnibus ANOVA Test** | | | | | |
| --- | --- | --- | --- | --- | --- |
|  | **Sum of Squares** | **df** | **Mean Square** | **F** | **p** |
| **Have you been trained to use the App?** | 5.83 | 1 | 5.832 | 13.76 | <.001 |
| **Add sNTDs surveillance features** | 6.30 | 1 | 6.299 | 14.86 | <.001 |
| **Perception about adding internet-dependent functions** | 12.20 | 4 | 3.051 | 7.20 | <.001 |
| **Residuals** | 73.34 | 173 | 0.424 |  |  |
| Note. Type 3 sum of squares | | | | | |

| **Table 3. Model Coefficients - App quality mean score** | | | | | | |
| --- | --- | --- | --- | --- | --- | --- |
|  | | | **95% Confidence Interval** | |  | |
| **Predictor** | **Estimate** | **SE** | **Lower** | **Upper** | **t** | **p** |
| Interceptᵃ | 3.7228 | 0.141 | 3.4448 | 4.001 | 26.433 | <.001 |
| Have you been trained to use the App?: |  |  |  |  |  |  |
| No – Yes | -0.3891 | 0.105 | -0.5962 | -0.182 | -3.709 | <.001 |
| Add sNTDs surveillance features: |  |  |  |  |  |  |
| No – Yes | -0.7257 | 0.188 | -1.0972 | -0.354 | -3.855 | <.001 |
| Perception about adding internet-dependent functions: |  |  |  |  |  |  |
| I mind it. Most of the time I do not have access to the internet on my phone – I mind a lot. I hardly never have internet connection on my phone | 0.0473 | 0.159 | -0.2656 | 0.360 | 0.298 | 0.766 |
| I do not care. Sometimes I have internet on my phone – I mind a lot. I hardly never have internet connection on my phone | -0.1666 | 0.245 | -0.6498 | 0.316 | -0.681 | 0.497 |
| I am up for it. Most of the time I have internet on my phone – I mind a lot. I hardly never have internet connection on my phone | 0.2600 | 0.153 | -0.0414 | 0.561 | 1.702 | 0.090 |
| I think it would be great. I always have internet on my phone – I mind a lot. I hardly never have internet connection on my phone | 0.9091 | 0.207 | 0.5008 | 1.317 | 4.395 | <.001 |
| ᵃ Represents reference level | | | | | | |

**Key findings from model coefficients**

- **Training to use the app**: Participants trained to use the app rated its quality significantly higher than those who were not trained (**β = -0.389, SE = 0.105, p < 0.001**).
- **Favoring the addition of surveillance features**: Those who supported adding surveillance features rated the app higher (**β = -0.726, SE = 0.188, p < 0.001**).
- **Perception of adding internet-dependent functions**:
  - Participants who **always have internet access** rated the app significantly higher than those with very limited internet (**β = 0.909, SE = 0.207, p < 0.001**).
  - Other comparisons within this category did not show significant differences (p > 0.05).

**Assumption checks.**

| **Table 4. Durbin–Watson test for autocorrelation.** | | |
| --- | --- | --- |
| **Autocorrelation** | **DW Statistic** | **p** |
| 0.0154 | 1.97 | 0.808 |

| **Table 5. Collinearity statistics.** | | |
| --- | --- | --- |
|  | **VIF** | **Tolerance** |
| **Have you been trained to use the app?** | 1.02 | 0.977 |
| **Add sNTDs surveillance features** | 1.00 | 0.996 |
| **Perception of adding internet-dependent functions** | 1.01 | 0.995 |

| **Table 6. Normality test (Shapiro-Wilk).** | |
| --- | --- |
| **Statistic** | **p** |
| 0.995 | 0.767 |

**Assumption checks**

- **Autocorrelation**: The **Durbin-Watson statistic (1.97, p = 0.808)** indicates no significant autocorrelation.
- **Collinearity**: The **Variance Inflation Factor (VIF) values** are close to 1, confirming no multicollinearity issues.
- **Normality**: The **Shapiro-Wilk test (p = 0.767)** suggests that residuals are normally distributed.

**Purpose of the second analysis – model 2**

In this regression analysis examined the statistical association between the app quality mean score and independent variables not included in its calculation:

1. Participant has undergone training to use the app
2. Participant in favor of the addition of surveillance features
3. Participant in favor of the addition of internet-dependent functions
4. Age
5. Sex
6. Type of survey participant
7. Participant interested for a desktop version of the app
8. Participant’s work context
9. Region
10. Participant’s frequency of dealing with patients with skin diseases
11. Dermatology experience
12. How participants heard about the app
13. Participant in favor of the addition of a data saving option for patients records
14. Participant in favor of translating the app
15. How many times the participant intend to use the app in upcoming 12 months
16. App’s likelihood to increase participant’s knowledge about skin NTDs
17. Willingness to recommend the app
18. Willingness to pay for the app

This analysis aimed to determine whether these factors significantly influence user perception of app quality. While running the model, we progressively removed unsignificant variables from the model.

| Model Fit Measures | | | | | | | |
| --- | --- | --- | --- | --- | --- | --- | --- |
|  | | | | **Overall Model Test** | | | |
| **Model** | **R²** | **Adjusted R²** | **AIC** | **F** | **df1** | **df2** | **p** |
| 1 | 0.450 | 0.431 | 305 | 23.6 | 6 | 173 | <.001 |
| Note. Models estimated using sample size of N=180 | | | | | | | |

**Model fit and significance**

- Predictors explain **45%** of variance in app quality scores (**R² = 0.450, adjusted R² = 0.430**), indicating a moderate explanatory power.
- The **F-test** is statistically significant (**F(6,173) = 23.6, p < 0.001**), suggesting that at least one of the predictor variables contributes significantly to the model.

| **Table 2. Omnibus ANOVA test.** | | | | | |
| --- | --- | --- | --- | --- | --- |
|  | **Sum of Squares** | **df** | **Mean Square** | **F** | **p** |
| **Have you been trained to use the App?** | 1.59 | 1 | 1.589 | 5.25 | **0.023** |
| **This app is likely to increase my knowledge about skin NTDs (Perceived impact 2)** | 6.49 | 1 | 6.493 | 21.43 | **<.001** |
| **Would you recommend this app to people who might benefit from it?** | 5.07 | 1 | 5.072 | 16.74 | **<.001** |
| **How many times do you think you would use this app in the next 12 months?** | 1.93 | 1 | 1.932 | 6.38 | **0.012** |
| **Do you have experience in dermatology?** | 2.13 | 2 | 1.064 | 3.51 | **0.032** |
| **Residuals** | 52.42 | 173 | 0.303 |  |  |
| Note. Type 3 sum of squares | | | | | |

Finally, only five variables were retained as significant in the model.

| **Table 3. Model Coefficients - App quality mean score** | | | | | | |
| --- | --- | --- | --- | --- | --- | --- |
|  | | | **95% Confidence Interval** | |  | |
| **Predictor** | **Estimate** | **SE** | **Lower** | **Upper** | **t** | **p** |
| Interceptᵃ | 1.3637 | 0.2418 | 0.8864 | 1.8410 | 5.639 | <.001 |
| Have you been trained to use the App?: |  |  |  |  |  |  |
| No – Yes | -0.2022 | 0.0883 | -0.3764 | -0.0280 | -2.290 | **0.023** |
| This app is likely to increase my knowledge about skin NTDs (Perceived impact 2) | 0.2883 | 0.0623 | 0.1654 | 0.4113 | 4.629 | **<.001** |
| Would you recommend this app to people who might benefit from it? | 0.1709 | 0.0418 | 0.0885 | 0.2534 | 4.092 | **<.001** |
| How many times do you think you would use this app in the next 12 months? | 0.1252 | 0.0496 | 0.0273 | 0.2230 | 2.525 | **0.012** |
| Do you have experience in dermatology?: |  |  |  |  |  |  |
| Not trained but experienced – Not trained & not experienced | 0.0172 | 0.0880 | -0.1566 | 0.1909 | 0.195 | 0.846 |
| Trained or experienced – Not trained & not experienced | 0.4653 | 0.1779 | 0.1142 | 0.8164 | 2.616 | **0.010** |
| ᵃ Represents reference level | | | | | | |

**Assumption Checks**

| Durbin–Watson Test for Autocorrelation | | |
| --- | --- | --- |
| **Autocorrelation** | **DW Statistic** | **p** |
| 0.0609 | 1.87 | 0.340 |

| **Collinearity Statistics** | | |
| --- | --- | --- |
|  | **VIF** | **Tolerance** |
| **Have you been trained to use the App?** | 1.02 | 0.982 |
| **This app is likely to increase my knowledge about skin NTDs (Perceived impact 2)** | 1.23 | 0.811 |
| **Would you recommend this app to people who might benefit from it?** | 1.26 | 0.794 |
| **How many times do you think you would use this app in the next 12 months?** | 1.24 | 0.805 |
| **Do you have experience in dermatology?** | 1.02 | 0.976 |

| **Normality Test (Shapiro-Wilk)** | |
| --- | --- |
| **Statistic** | **p** |
| 0.995 | 0.782 |

**Interpretation of assumption checks**

- **Autocorrelation**: The **Durbin-Watson statistic (1.87, p = 0.340)** indicates no significant autocorrelation.
- **Collinearity**: The **Variance Inflation Factor (VIF) values** are close to 1, confirming no multicollinearity issues.
- **Normality**: The **Shapiro-Wilk test (p = 0.782)** suggests that residuals are normally distributed.

**Key findings from model coefficients**

- Participants **trained to use the app** rated its quality significantly higher than those who were not trained (**β = -0.202, SE = 0.088, p = 0.023**).
- App quality mean score increased with users’ perception that **the app is likely to increase their knowledge about skin NTDs** (**β = 0.288, SE = 0.062, p < 0.001**).
- Participants’ willingness to recommend the app was associated with higher app’s mean quality score (**β = 0.171, SE = 0.042, p < 0.001**).
- App quality mean score increased with the number of times a user planned to use the app in next 12 months (**β = 0.125, SE = 0.500, p = 0.012**).
- There was a difference in app’s quality mean score among participants trained or experienced in dermatology vs those not trained and not experienced (**β = 0.465, SE = 0.178, p = 0.010**).
