# Supplementary material for: Usability and quality evaluation of the World Health Organization SkinNTDs app among frontline health workers in Cameroon: A mixed methods study
Source: PLoS Negl Trop Dis. 2025 Sep 10;19(9):e0013461. doi: 10.1371/journal.pntd.0013461 (PMC12422481; doi:10.1371/journal.pntd.0013461)
Supplement: S5 Appendix — (DOCX) [file pntd.0013461.s005.docx]

**Supporting information file.**

**S5 Appendix. Results of qualitative data analysis.**

The suggestions and comments made in the questionnaires and focus groups were thematically analyzed. Major themes and sub-themes that align with the uMARS framework are shown in Table 1 along with the most illustrative quotes.

**PART 1: Thematic analysis**

**Table 1. Themes aligned with the uMARS framework and illustrative quotes.**

| **Major Themes** | **Sub-Themes** | **Selected Quotes** |
| --- | --- | --- |
| **Domain: 1. *Engagement*** | | |
| Appeal and interest in the app. | Learning and continuous education. | *"The app is a great tool for learning about skin NTDs, especially for diseases we don’t see often."* |
| Motivation to use the app regularly. | Gamification and interactive features (e.g., quizzes, case studies). | *"Adding quizzes or case studies would make the app more engaging and fun to use."* |
| **Domain: 2. *Functionality*** | | |
| Ease of use and navigation. | Simplified interface for non-tech-savvy users. | *"The app is easy to navigate, but some of my colleagues struggle with smartphones. A simpler design would help."* |
| Technical performance (e.g., loading times, crashes). | Offline functionality for areas with poor internet access. | *"The app needs to work offline. In rural areas, we often don’t have internet access."* |
| Customization options (e.g., font size, brightness). | Search function and intuitive navigation. | *"I wish I could adjust the font size. The text is too small for me to read comfortably."* |
| **Domain: 3. *Aesthetics*** | | |
| Visual appeal and layout. | High-quality images and graphics. | *"The images are very clear and helpful for diagnosing skin conditions."* |
| Image quality and relevance. | Use of vibrant colors and interactive elements. | *"The app looks good, but it could use more colors to make it more visually appealing."* |
| **Domain: 4. *Information Quality*** | | |
| Accuracy and reliability of information. | Detailed treatment protocols and posology. | *"The treatment guidelines are accurate, but I’d like to see more details on dosages."* |
| Relevance to local context (e.g., images of darker skin tones). | Regular updates to reflect latest guidelines and research. | *"We need more images of darker skin tones. Many of the current images don’t match what we see in our patients."* |
| Comprehensiveness of content. | Localization (e.g., translations into local languages). | *"The app should be available in Hausa and Fulani. Not everyone understands English."* |
| **Domain: 5. *Subjective Quality*** | | |
| Overall satisfaction with the app. | Perceived value as a training and diagnostic tool. | *"This app is a game-changer for diagnosing skin NTDs in remote areas."* |
| Recommendations for improvement. | Concerns about over-reliance on the app for diagnosis. | *"We need to be careful not to rely too much on the app. It should support, not replace, our clinical skills."* |

As shown above, participants particularly praised the educational value of the app and ease of use but suggested such improvements as localized content and more interactive features to enhance engagement. Major themes and sub-themes that do not align with the uMARS framework are shown in Table 2 along with the most illustrative quotes.

**Table 2. Themes not aligned with the uMARS framework and illustrative quotes.**

| **Major Themes** | **Sub-Themes** | **Selected Quotes** |
| --- | --- | --- |
| **Category: 1. Training and Capacity Building** | | |
| Language in the app adapted to context and users. | Language and literacy barriers (e.g., simpler language, local translations). | *"Many health workers in rural areas don’t speak English. The app needs to be in local languages."* |
| Need for training on skin NTDs and app usage. | Continuous learning and refresher courses. | *"We need regular training sessions to keep up with new features and updates in the app."* |
| **Category: 2. Integration into Healthcare Systems** | | |
| Challenges of over-reliance on the app. | Balancing app use with clinical skills and autonomy | *"If we rely too much on the app, we might lose our ability to diagnose without it."* |
| Conditions for integrating the app within the health system. | Government endorsement and support. | *"The government should officially endorse this app and include it in national health programs."* |
|  | Training programs for health workers. | *"We need training sessions to teach health workers how to use the app effectively."* |
|  | Availability of devices and internet access. | *"The government should provide smartphones and internet access to health workers in rural areas."* |
| The ideal app user. | Frontline health workers (e.g., nurses, community health workers). | *"This app is perfect for nurses and community health workers who don’t have access to dermatologists."* |
|  | Medical students and trainees. | *"Medical students can use this app to learn about skin NTDs and practice their diagnostic skills."* |
|  | Dermatologists and specialists for reference use. | *"Even specialists can use it as a quick reference tool, especially in remote areas."* |
| Main challenges to app use. | Lack of internet access in remote areas. | *"In rural areas, we often don’t have internet. The app needs to work offline."* |
|  | High cost of smartphones and data. | *"Many health workers can’t afford smartphones. This limits who can use the app."* |
|  | Limited technical skills among older health workers. | *"Some of my colleagues struggle with smartphones. They need training to use the app."* |
| Most beneficial time for app use. | During patient consultations for real-time diagnosis. | *"I use the app during consultations to confirm my diagnosis and check treatment options."* |
|  | During training sessions or refresher courses. | *"The app is great for training. We use it during workshops to teach about skin NTDs."* |
|  | During free time for continuous learning. | *"I review the app in my free time to stay updated on skin NTDs. It’s like a mini-course."* |
| **Category: 3. Dissemination and Promotion** | | |
| Strategies for promoting the app (e.g., training workshops, social media). | Use of WhatsApp and social media for dissemination. | *"We should share the app on WhatsApp groups. That’s how most health workers communicate."* |
| Challenges in dissemination (e.g., cost of devices, lack of internet access). | Government support for providing devices to health workers. | *"Many health workers can’t afford smartphones. The government should provide devices to those in need."* |
| **Category: 4. Cultural and Contextual Considerations** | | |
| Local relevance (e.g., images of darker skin tones, local languages). | Cultural sensitivity and adaptation to local beliefs and practices. | *"The app should include more images of African skin tones. Many of the current images don’t match our patients."* |
| Community engagement (e.g., training for community health workers). | Contextual adaptation for urban vs. rural settings. | *"Community health workers should be trained to use the app. They are the first point of contact for many patients."* |
| **Category: 5. Technical and Practical Challenges** | | |
| Device compatibility and accessibility. | Electricity and connectivity issues (e.g., solar chargers, power banks). | *"In rural areas, we often don’t have electricity to charge our phones. Solar chargers would help."* |
| Cost of devices and data. | Storage and performance issues (e.g., app size, slow loading). | *"The app takes up too much space on my phone. I can’t use it because I don’t have enough storage."* |
| Maintenance and updates. | Regular updates to address bugs and improve performance. | *"The app needs regular updates to fix bugs and improve performance."* |
| **Category: 6. Ethical and Social Considerations** | | |
| Patient privacy and confidentiality. | Informed consent for using patient data. | *"We need to ensure patient data is protected when using the app."* |
| Equity and access (e.g., digital divide). | Addressing health inequities in resource-limited settings. | *"Not everyone has access to smartphones. This could create a gap between those who can use the app and those who can’t."* |

**PART 2:** **Key comments and suggestions from participants gathered through online questionnaire.**

**Content of training if received:**

- P01: Training on skin-related NTDs, leishmaniasis, leprosy, yaws, buruli ulcer
- P02: Diagnosis and management of different NTDs
- P07: Skin diseases
- P08: Management of NTDs
- P12: Leprosy, yaws, leishmaniasis
- P13: Community management of lymphedema
- P17: Onchocerciasis, lymphatic filariasis and leprosy
- P18: Diagnosis and management
- P21: Causes, pathogen, management
- P25: Signs and symptoms, diagnosis and management
- P33: NTDS
- P41: Diagnosis and management of skin-related NTDs
- P72: Identification of NTDs
- P81: Treatment of the following diseases: Onchocerciasis, yaws, lymphatic filariasis, hydrocele, intestinal worms, bilharzia
- P91: Diagnosis, manifestations and treatment of NTDs
- P98: On neglected tropical diseases, oncho, filariasis etc.
- P105: Cutaneous leishmaniasis
- P108: NTDs causes/signs/symptoms and means of management
- P121: Aspects of epidemiological surveillance of NTDs

**Suggestion – Engagement section**

- P13: Add other features including geographic references
- P17: App should be free to allow continued use
- P26: Always continue to improve, for other pathologies as well
- P27: Continue in the same vein as it is very interesting
- P34: Initiate training
- P41: Provide option of asking more complex questions through internet link to a specialist
- P45: Add all NTDs
- P48: Requiring an internet connection is a restriction for users.
- P52: More prescriptions
- P53: The application must not be negligible
- P55: Training needed for each app to strengthen our capacities in this area
- P57: Train users in dermatology
- P58: Organize specific training
- P60: Accompany presentations with video
- P65: Provide more knowledge.
- P68: Users will need to be trained on the app
- P72: Training
- P73: Training for providers
- P74: Need training on care and making a correct diagnosis
- P75: Improve interactive features
- P76: We like the app before we actually know it. It should be the other way around.
- P79: Incomplete translation of words and sentences
- P82: Make it more efficient, especially with definitions and color images/photos of patients
- P84: Very important for knowledge about NTDs
- P85: Update the application
- P87: Provide excellent training on use of the app
- P102: Enable the addition and sharing of experiences
- P105: Increase the number of pathologies
- P106: Improve the app
- P110: Make the app more adequate
- P112: Oral questions are needed
- P118: Provide training in order to have experts in the field of MTN diseases
- P119: Improve image quality
- P125: Does the app prescribe medication?
- P126: Access should be free
- P28: Talk about patients in general
- P129: More details on follow-up
- P133: Make images more visible
- P134: More images of conditions
- P135: More customization options and feedback possibilities to improve contents
- P142: Talk about pathologies other than dermatology
- P143: Expand glossary section to include more definitions
- P144: Enable customization of brightness and font size, and make more interactive
- P150: What are the advantages of this app?
- P153: What is the importance of this app?
- P165: Clearly specify the importance of the app in the questionnaire
- P181: Allow viewing of all conditions without connection

**Suggestion - Functionality section**

- P13: Improve links between tables
- P17: Simplify questions
- P45: Make app easier to use for caregivers in the field with little experience of phones
- P52: Improve performance
- P53: Provide more information on app features
- P57: Train users on this software
- P58: Organize specific training
- P65: Provide more field knowledge
- P68: Unstable connection
- P72: A support
- P55: Training
- P74: Need for internet credit
- P76: More specificity in cases
- P82: Explain the images
- P87: Insufficient network to use the application
- P105: Reset certain features
- P106: Provide a lot of images
- P110: Make the app easy to use
- P112: Make questions understandable
- P119: the mobility (interactivity, navigability) of the images
- P120: Very good app
- P126: Everything is ok
- P128: Have checkboxes in the app
- P129: Manage negligible problems
- P133: Review the splash images, the loading is slow
- P134: Difficult to scroll through images
- P135: Make it more efficient
- P143: Add more features
- P144: Improve interaction
- P147: Precise functionality
- P160: It is too complicated
- P161: Understood
- P169: Very good app
- P179: Improve the questionnaire
- P180: Adequate features

**Suggestion - Aesthetics section**

- P13: Update and improve the presentation of graphics
- P17: Simplify further
- P33: Increase font size
- P43: The characters are small
- P44: The pictures are small
- P45: Make it more memorable
- P50: Good enough
- P52: Improve the graphics
- P57: Train users on this software
- P58: Organize specific training
- P65: Mastery of the tool
- P68: Not mastered
- P72: Support
- P73: Improvement
- P76: Strengthen the sharpness of photos
- P87: Strengthen user capacity
- P91: Train how to use icons
- P106: Increase the visual appeal
- P110: Graphics and visual design must be clear
- P112: Questions must be easy
- P119: Improve the quality of graphics
- P120: Very good application
- P126: Everything is OK
- P129: Use high quality graphics
- P133: This part must be corrected
- P135: Have a variety of colors according to the cases of NTDs
- P137: Improve the app
- P138: Very good
- P143: Improve the aesthetics to make it more attractive
- P144: Readjust the stylistic consistency
- P151: Not convincing
- P160: It is a bit difficult
- P169: Good app
- P180: Very aesthetic

**Suggestions for Information section**

- P13: Government endorsement needed
- P17: Too long
- P45: This app should be developed by the government
- P52: Have more diagnostics in dermatology
- P57: Train users on this software
- P58: Organize specific training
- P65: Opening the user tool
- P68: Simplify things
- P72: Support
- P73: Improvement
- P74: Please train us
- P82: Improve
- P87: Briefing on the application at any time
- P94: Explicit
- P110: Components must be clear
- P112: Questions must be well asked
- P119: Improve the resolution of the app
- P120: Good app
- P129: Improve images
- P133: Must be reliable
- P133: No worries
- P144: Improve features
- P147: Satisfactory
- P151: Makes user lazy
- P160: Not understandable
- P161: Good
- P180: Clear information

**Suggested languages**

- P07: Arabic, Hausa
- P08: Fulfulde
- P10: Many other languages
- P12: Mafa, Fulfulde
- P13: English, French
- P14: English
- P17: Ewondo, Fulfulde
- P26: Moundang
- P29: Fulfulde, Toupouri
- P31: Arabic
- P34: Mafa
- P70: Fulfulde, Arabic
- P76: Fulfulde, Musgum
- P77: Haoussa, Fulfulde, Mboum, Baya
- P79: Laka, Fulfulde
- P81: English, Haoussa, Arabic, Fulfulde
- P83: Local languages
- P91: Espagnol, Fulfulde, Tupuri
- P96: Spanish
- P102: German, Spanish, Chinese, Russian
- P103: Toupouri
- P110: English and Fulfuldé
- P112: Foulbe, Chinese
- P114: English, Spanish, German, Arabic
- P120: French
- P121: Fulbe
- P123: Guiziga
- P127: Chinese, Spanish, Arabic, German, Portuguese
- P128: Toupouri, Massa
- P130: Fulfulde, Spanish and German
- P132: Mboum
- P136: Toupouri and Massa
- P140: Chinese, Spanish
- P145: The various languages of the Country
- P146: German
- P149: Chinese
- P166: German and Spanish
