# Supplementary material for: Usability and quality evaluation of the World Health Organization SkinNTDs app among frontline health workers in Cameroon: A mixed methods study
Source: PLoS Negl Trop Dis. 2025 Sep 10;19(9):e0013461. doi: 10.1371/journal.pntd.0013461 (PMC12422481; doi:10.1371/journal.pntd.0013461)
Supplement: S1 Table — (DOCX) [file pntd.0013461.s006.docx]

**Supporting information file.**

**S1 Table. Details of participants in focus group sessions.**

| **Session number** | **Region/place** | **Number of participants*** | **Profile of participants** |
| --- | --- | --- | --- |
| 1 | North (Garoua public state-registered nurse training school) | 58 (27F, 31M) | Third-year students |
| 2 | North (Garoua private state-registered nurse training school) | 78 (39F, 39M) | Third-year students |
| 3 | Far North (Maroua public state-registered nurse training school) | 29 (26F, 3M) | Third-year students |
| 4 | Far North (Maroua 3 Health District) | 16 (3F, 13M) | Chiefs of health areas, data manager at district level, supervisor from regional delegation |
| 5 | Far North (Roua Health District) | 15 (3F, 12M) | Chiefs of health areas, data manager at district level |
| 6 | North (Lagdo Health District) | 18 (2F, 16M) | Chiefs of health areas, data manager at district level |
| Summary | 6 sessions with 214 participants (100 Females, 114 Males) | | |

*M = Males, F = Females
