# Supplementary material for: Usability and quality evaluation of the World Health Organization SkinNTDs app among frontline health workers in Cameroon: A mixed methods study
Source: PLoS Negl Trop Dis. 2025 Sep 10;19(9):e0013461. doi: 10.1371/journal.pntd.0013461 (PMC12422481; doi:10.1371/journal.pntd.0013461)
Supplement: S2 Table — (DOCX) [file pntd.0013461.s007.docx]

**Supporting information.**

**S3 Table. Deviations from the study protocol.**

| **Planned approach described in the protocol** | **Type, description and justification of the deviation from the protocol** |
| --- | --- |
| We planned to only include frontline heath workers (FHWs). | Type of deviation: addition  We decided to include third-year students in state-registered nurse training schools as we believed these could serve as a proxy for FHWs. In addition, third-year students are expected to be operational FHWs soon after graduation.  We also added a questionnaire specifically to capture the viewpoints of key informants at strategic level within the NTDs program. |
