# Supplementary material for: Usability and quality evaluation of the World Health Organization SkinNTDs app among frontline health workers in Cameroon: A mixed methods study
Source: PLoS Negl Trop Dis. 2025 Sep 10;19(9):e0013461. doi: 10.1371/journal.pntd.0013461 (PMC12422481; doi:10.1371/journal.pntd.0013461)
Supplement: S1 Dataset — (ZIP) [file pntd.0013461.s008.zip › Data files/Focus group data.docx]

**DATA FILE: FOCUS GROUP DISCUSSIONS NOTES TRANSCRIPTION.**

**Focus Group Session 1.**

| **Num** | | **Questions** | | **Réponses** |
| --- | --- | --- | --- | --- |
| 1 | | Quelles fonctionnalités pensez-vous essentielles à l’application ? Quelles sont celles que vous ne changeriez-pas. Pourquoi ? | Fonctionnalités identifiez : langue changeable, accueil, menu, champ de recherche dans la partie diagnostique.  L’application répond facilement aux questions.  Faible consommation de données internet.  Garder la fonctionnalité Diagnostic : si on identifie les signes et symptômes mais on n’a pas de diagnostic déclaré cela ne sert à rien.  La fonctionnalité Index mondial : permet de connaitre le nombre de cas de MTN du pays.  Ne supprimer aucune fonctionnalité. | |
| 2 | | Quelles fonctionnalités pensez-vous les moins essentielles ? Quelles sont celles que vous enlèveriez. Pourquoi ? | Index Mondial pourrait ne pas être nécessaire car l’application est censée servir d’outil de diagnostic. Elle devrait donner des indications sur des échelles plus réduites telles que les localités, pas seulement au niveau global pays. | |
| 3 | | Quelles fonctionnalités devraient absolument être dans l’application pour que vous l’utilisiez ou que vous la recommandiez ? | Diagnostic | |
| 4 | | Entrevoyez-vous que l’application puisse être intégrée comme un dispositif médical standard dans votre service ? Pourquoi ? | Oui : L’application pourrait aider dans le diagnostic dans les services de dermatologie.  Non : les patients pourraient ne pas apprécier que les cliniciens utilisent une application mobile en leur présence pour effectuer un diagnostic. Tout patient pourrait télécharger l’application et s’auto-diagnostiquer sans plus recourir aux services de santé/cliniciens. Les cliniciens deviendraient inutiles. « *Non :* *il n’est pas sûr que tous les prestataires auraient un smartphone pour installer l’application*. »  Non : « *dans l’urgence, un clinicien ne va utiliser son téléphone pour poser le diagnostic d’un patient* ». Cela pourrait contribuer a une crise de confiance patient-soignant.  Non : l’application pourrait rendre le personnel paresseux. Ces derniers pourraient ne plus etre capables prendre en charge des patients sans leur telephone.  Non : en cas de dysfonctionnement de l’application, il pourrait y avoir cessation de service dans les services de sante.  « c’est une application numerique, il y a toujours des bug. Elle pourrait etre piratee. »  Non : la medecine est dynamique. Disponibiliser l’application c’est rendre la medicine statique.  Oui : application en elle-meme vient renforcer les connaissances et non pas remplacer les prestataires. C’est une formation continue. | |
| 5 | | Utilisez-vous une autre application mobile de santé dans votre pratique quotidienne? Et vos collègues ? | Flo, Medscape, Epocrates | |
| 6 | | Après avoir utilisé l’application, qui pensez-vous qui en soit l’utilisateur final idéal ? | Apprenants et personnels soignants : en tant qu’apprenant, l’application m’a appris beaucoup de choses.  Infirmier : dans un CSI, l’infirmier peut diagnostiquer sur la base des signes et symptômes et référer chez le spécialiste pour une meilleure prise en charge.  Tout clinicien consultant, tout relais communautaire : les relais communautaires sont en contact avec certains patients en communautés.  Confier l’application aux prestataires suivant l’environnement (zones rurales : infirmiers, aide soignants, relais communautaires) et la formation sanitaire. | |
| 7 | | Quelle serait la Meilleure façon de disséminer l’application ? | Le DRSP devrait organiser un seminaire de formation a l’intention de tous les responsables des formations sanitaires ;  Organiser des seminaires de formation pour les personnels soignants idealement en presentiel car les zones avec acces internet limite pourraient ne pas suivre correctement. | |
| 8 | | Pensez-vous que 5 jours soient suffisants pour tester l’application et être capable de répondre au questionnaire MARS ? | Oui, on apprend vite | |
| 9 | | Autres | Fusionner les modules « Signes et symptomes » et « Diagnostic ».  10 participants ont obtenu l’application par Xender app. | |

**Focus Group Session 2.**

| **Num** | **Questions** | **Réponses** |
| --- | --- | --- |
|  | Quelles fonctionnalités pensez-vous essentielles à l’application ? Quelles sont celles que vous ne changeriez-pas. Pourquoi ? | Fonctionnalites identifiez : langue changeable, accueil, menu, champ de recherche dans la partie diagnostique.  L’application repond facilement aux questions.  Faible consommation de donnees internet.  Garder la fonctionalite Diagnostic : si on identifie les signes et symptomes mais on n’a pas de diagnostic declare cela ne sert a rien.  La fonctionalite Index mondial : permet de connaitre le nombre de cas de MTN du pays.  Ne supprimer aucune fonctionalite. |
|  | Quelles fonctionnalités pensez-vous les moins essentielles ? Quelles sont celles que vous enlèveriez. Pourquoi ? | Index Mondial pourrait ne pas etre necessaire car l’application est censee servir d’outil de diagnostic. Elle devrait donner des indications sur des echelles plus reduites telles que les localites, pas seulement au niveau global pays. |
|  | Quelles fonctionnalités devraient absolument être dans l’application pour que vous l’utilisiez ou que vous la recommandiez ? | La fonctionalite emplacement permet d’orienter le diagnostic differentiel  La fonctionnalite MTN a affections cutanees guide le diaqgnostique et la prise en charge des MTN a affections cutanees  La fontionnalite chatbot est a retenir et ameliorer |
|  | Entrevoyez-vous que l’application puisse être intégrée comme un dispositif médical standard dans votre service ? Pourquoi ? | Oui : L’application pourrait aider dans le diagnostic dans les services de dermatologie.  Non : les patients pourraient ne pas apprecier que les cliniciens utilisent une application mobile en leur presence pour effectuer un diagnotic. Tout patient pourrait telecharger l’application et s’auto-diagnostiquer sans plus recourir aux services de sante/cliniciens. Les cliniciens deviendraient inutiles. Non : il n’est pas sur que tous les prestataires auraient une smartphone pour installer l’application.  Non : dans l’urgence, un clinicien ne vas utiliser son telephone pour poser le diagnostic d’un patient. Cela pourrait contribuer a une crise de confiance patient-soignant.  Non : l’application pourrait rendre le personnel paresseux. Ces derniers pourraient ne plus etre capables prendre en charge des patients sans leur telephone.  Non : en cas de dysfonctionnement de l’application, il pourrait y avoir cessation de service dans les services de sante.  « c’est une application numerique, il y a toujours des bug. Elle pourrait etre piratee. »  Non : la medecine est dynamique. Disponibiliser l’application c’est rendre la medicine statique.  Oui : application en elle-meme vient renforcer les connaissances et non pas remplacer les prestataires. C ;est une formation continue. |
|  | Utilisez-vous une autre application mobile de santé dans votre pratique quotidienne? Et vos collègues ? | Encarta |
|  | Après avoir utilisé l’application, qui pensez-vous qui en soit l’utilisateur final idéal ? | Apprenants et personnels soignants : en tant qu’apprenant, l’application m’a appris beaucoup de choses.  Infirmier : dans un CSI, l’infirmier peut diagnostiquer sur la base des signes et symptomes et referer chez le specialiste pour une meilleure prise en charge.  Tout clinicien consultant, tout relais communautaire : les relais communautaires sont en contact avec certains patients en communautes.  Confier l’application aux prestataires suivant l’environnement (zones rurales : infirmiers, aide soignants, relais commautaires) et la formation sanitaire. |
|  | Quelle serait la Meilleure façon de disséminer l’application ? | Le DRSP devrait organiser un seminaire de formation a l’intention de tous les responsables des formations sanitaires ;  Organiser des seminaires de formation pour les personnels soignants idealement en presentiel car les zones avec acces internet limite pourraient ne pas suivre correctement. |
|  | Pensez-vous que 5 jours soient suffisants pour tester l’application et être capable de répondre au questionnaire MARS ? | Oui. C’est meme beaucoup. |
|  | Autres | Fusionner les modules « Signes et symptomes » et « Diagnostic ».  10 participants ont obtenu l’application par Xender app. |

**Focus Group Session 3.**

| **Num** | **Questions** | **Réponses** |
| --- | --- | --- |
| 1 | Quelles sont les fonctionnalités que vous avez identifiées dans l’application ? | Diagnostiquer les maladies de la peau.  Ressortie les signes et symptômes des MTN a affections cutanées. |
| 2 | Quelles fonctionnalités pensez-vous essentielles à l’application ? Quelles sont celles que vous ne changeriez-pas. Pourquoi ? | Signes et symptômes : parce que ça permet d’avoir une idée sur les signes lies à une maladie.  Diagnostic : facilite la tâche. Permet de poser un diagnostic fiable. Oriente pour la prise en charge.  Pour certaines maladies on n’a pas de traitement. |
| 3 | Quelles fonctionnalités pensez-vous les moins essentielles ? Quelles sont celles que vous enlèveriez. Pourquoi ? | On pourrait se passer du robot (chatbot) car il parle des diagnostics déjà dans d’autres fonctionnalités de l’application.  Index Mondial : car il n’y a pas de données pour plusieurs pathologies. On ne sait pas à quoi ça sert. |
| 4 | Quelles fonctionnalités devraient absolument être dans l’application pour que vous l’utilisiez ou que vous la recommandiez ? | Diagnostic et Signes et symptômes, là où il y a les images.  Les images peuvent nous aider à reconnaitre une maladie qu’on a vu. |
| 5 | D’après votre compréhension, qu’est-ce que cette application pourrait faire pour vous ? | Nous aider à poser un diagnostic.  Nous aider comme aide-mémoire pour les examens.  Nous aider à confirmer notre diagnostic à travers les images.  Aider à identifier les cas de MTN |
| 6 | Si vous deviez utiliser l’application ou si un personnel de sante en service dans une formation sanitaire devait utiliser l’application, quel serait le meilleur moment pour utiliser l’application ? | Face à un cas devant nous, si on ne connait pas le diagnostic on utilise l’application pour nous aider.  Au moment de la consultation.  En cas de doute. On peut aussi entrer dans l’application pour confirmer le diagnostic et éviter l’ignorance.  Il faut tenir compte du patient que nous avons devant nous. On pourrait se déplacer hors de la vue du patient et aller regarder l’application.  Elle a raison. Mais sur le terrain on sait que si le personnel de sante est dépassé, il appelle au téléphone pour demander conseil. Donc on peut aussi utiliser l’application en présence du patient ! |
| 7 | Entrevoyez-vous que l’application puisse être intégrée comme un dispositif médical standard dans votre service ? Pourquoi ? | C’est possible. « Sur le terrain c’est compliqué. On n’a pas de dermatologue. Donc si on n’a pas de dermatologue et qu’on peut utiliser l’application. »  Si c’est possible, insérer d’autres pathologies dans l’application.  Non. Ça serait d’imprimer les signes et symptômes et images comme des affiches et les afficher dans les formations sanitaires plutôt que d’utiliser le téléphone.  On pourrait rendre l’application obligatoire car on a du mal à retenir beaucoup de choses.  On ne peut pas reprendre cela obligatoire car ce n’est pas tout le monde qui a un téléphone androïde.  « Est-ce que l’OMS va offrir un téléphone a tout le monde ? dans ce cas, on peut rendre l’application obligatoire ». |
| 8 | Utilisez-vous une autre application mobile de santé dans votre pratique quotidienne ? Et vos collègues ? | Oui : manuel sante ; Gestogramme ; Pharmacologie ; maladies infectieuses ; Prescriptions ; Quiz médical ; Pédiatrie ; Gynécologie ; |
| 9 | Après avoir utilisé l’application, qui pensez-vous qui en soit l’utilisateur final idéal ? | Le médecin ; le Dermatologue ; « celui qui consulte, même si c’est un infirmier ou un AS. »  Le dermatologue n’est pas forcement expert. Il peut aussi avoir besoin de l’application. |
| 10 | Quelle serait la Meilleure façon de disséminer l’application ?  Comment proposeriez-vous de procéder pour que le maximum de personnes utilisateurs final idéal puissent être au courant de l’application et puisse l’installer ? | Envoyer les liens dans les groupes.  Il faut sensibiliser dans les centres de santé. Envoyer un délégué pour aller sensibiliser dans les centres de santé. C’est difficile de parcourir les centres de santé. |
| 11 | Pensez-vous que 5 jours soient suffisants pour tester l’application et être capable de répondre au questionnaire MARS ? | Oui. Ça devrait aller |
| 12 | Suggestions | Pourrait-on rendre disponible hors connexion la fonctionnalité du robot ?  On pourrait améliorer le glossaire sur les définitions des pathologies.  Pour les pathologies pour lesquelles l’application demande de référer le cas, ce serait de proposer une prise en charge locale en attendant que de référer le cas.  Ajouter d’autres signes pathognomoniques qui améliorent le diagnostic différentiel. |

**Focus Group Session 4.**

| **Num** | **Questions** | **Réponses** |
| --- | --- | --- |
| **01** | Avez-vous déjà utilisé une autre application mobile de santé dans votre pratique quotidienne ? Si oui, laquelle ? | - Le Larousse médical, qui donne les définitions des termes médicaux - Waspito, qui donne le diagnostic des maladies cardio-vasculaires - Redcap ; le diagnostic de la tuberculose et la prise en charge - DSM5 ; la classification des différentes pathologies - Med Index ; les signes les symptômes et le traitement de différentes maladies - Gestogramme ; qui donne l'âge de la grossesse et la date probable d'accouchement |
| **02** | Quelle est votre opinion générale de WHO SkinNTDs app ? D’après votre compréhension, qu’est-ce que cette application pourrait faire pour vous ? | - L'application permet de faire le diagnostic différentiel des maladies de la peau car on a plusieurs maladies dans l'application et lorsque on clique sur les signes et symptômes, ça nous renvoie spécifiquement à une maladie ce qui permet de faire le diagnostic différentiel entre plusieurs maladies - L'application permet de faire la prise en charge grâce à la partie management où on donne la molécule à prescrire au malade, comment faire le suivi et à quel moment référé le patient - L’application est un outil de recyclage parce qu'il y a les définitions de certains termes qui concernent les maladies de la peau desquelles nous avons entendu parler depuis longtemps avec les images qui les démontrent ; les affections de la peau qu’on aurait oubliées ou bien qu’on n’ait pas encore rencontré |
| 1 | Quelles sont les fonctionnalités que vous avez identifiées dans l’application ? | - Les fonctionnalités signes et symptômes, les images, le traitement et le suivi : cela permet de réduire la réflexion - On a les images dans l'application qui permettent de comparer le patient qu'on voit et les images qu'on a dans l'application. |
| 2 | Quelles fonctionnalités pensez-vous essentielles à l’application ? Quelles sont celles que vous ne changeriez-pas. Pourquoi ? | - Les fonctionnalités signes et symptômes, Diagnostic et Traitement : l'emplacement des signes et symptômes permet de poser un bon diagnostic car on peut comparer le patient et les images dans l'application |
| 3 | Quelles fonctionnalités pensez-vous les moins essentielles ? Quelles sont celles que vous enlèveriez. Pourquoi ? | - Aucune fonctionnalité n'est moins essentielle ou alors ne devrait être enlevé - On devrait enlever la partie où on explique la définition des termes (c'est-à-dire les signes et symptômes) n'est pas utile (1 avis sur 14) |
| 4 | Quelles fonctionnalités devraient absolument être dans l’application pour que vous l’utilisiez ou que vous la recommandiez ? | - On devrait ajouter à l'application un onglet pour télécharger les images et les petites définitions qui sont donnés pour les signes et symptômes. - On devrait mettre un onglet sur la page d'accueil pour faire la recherche des mots par exemple des maladies et des images directement. - Dans la partie management on devrait insérer la dénomination internationale des molécules pour la prise en charge avec des noms qui pourraient être facilement identifiables ou alors le nom commercial des produits qu'on doit prescrire au patient. |
| 5 | Si vous deviez utiliser l’application ou si un personnel de sante en service dans une formation sanitaire devait utiliser l’application, quel serait le meilleur moment pour utiliser l’application ? | - Le meilleur moment pour utiliser l'application c'est en salle de consultation en face du malade parce que on peut regarder le malade, les signes et symptômes qu’il présente et comparer avec les images qui sont dans l'application. - Le meilleur moment serait lors du temps libre du personnel ou alors pendant le temps de relaxation. - Pendant le temps libre on consulte l’application pour essayer de comparer les informations avec ce qu'on a vu sur un patient parce que cela permet de d'éviter que le patient pense qu'on lui manque de respect en utilisant son téléphone pendant qu'il est devant nous pour la consultation et aussi cela permettrait d'éviter que le patient sous-estime les compétences du consultant. - Le meilleur moment pour utiliser l'application serait pendant le renforcement des capacités avec les personnels de la formation sanitaire. |
| 6 | Entrevoyez-vous que l’application puisse être intégrée comme un dispositif médical standard dans votre service ? Pourquoi ? | - Non parce que ce n'est pas tout le personnel de la formation sanitaire qui est formé sur l'utilisation de l'application - Non à cause du problème d'électricité auquel font face plusieurs formations sanitaires dans les zones reculées - Non à cause du problème de réseau auquel font face plusieurs personnels dans certaines localités - Non parce qu’il faut un téléphone Android pour utiliser l'application et ce n'est pas tout le personnel de la formation sanitaire qui a un téléphone Android - Oui parce qu’on fait face à l'apparition de nouvelles maladies tous les jours |
| 7 | Après avoir utilisé l’application, qui pensez-vous qui en soit l’utilisateur final idéal ? | - Le médecin, l'infirmier et les spécialistes des maladies de la peau parce que ce sont ceux-ci qui sont en contact avec ces types de patients - Tout consultant est l'utilisateur idéal final car ce sont eux qui sont en constant contact avec les patients - Tous les personnels de santé et les étudiants pour leur propre connaissance - Toute personne cultivée c'est-à-dire quelqu'un qui a un téléphone Android, qui s'est lire et écrire et qui peut manipuler l'application. Ça peut l’aider à savoir de quoi il souffre et à savoir s'il faut aller à l'hôpital ou pas - Tous ceux qui aspire à se spécialiser en dermatologie |
| 8 | Quelle serait la Meilleure façon de disséminer l’application ?  Comment proposeriez-vous de procéder pour que le maximum de personnes utilisateurs final idéal puissent être au courant de l’application et puisse l’installer ? | - Le partage par WhatsApp par les liens accompagnés de captures d'écran d'images pour rendre cela captivant afin que celui qui reçoit puisse être amené à télécharger et à utiliser - L’OMS doit mobiliser des fonds pour organiser des séances de restitution accompagné d'un goûter ou d'une pause-café - Mettre l'application sur Play store - Faire des dons de tablette aux FOSA pour utiliser l'application - Partager dans des groupes WhatsApp avec les explications afin que les gens puissent télécharger |
| 9 | Quels défis entrevoyez-vous pour la dissémination, l’installation et l’utilisation de l’application ? Comment pensez-vous que ces défis puissent être adressés ? | - L'utilisation de l'application est un défi. Pour ceci il faut faire des descentes sur le terrain pour démontrer l'utilisation de l'application aux autres personnels - Le prix de l'installation est coûteux. Il faut mettre à la disposition des formations sanitaires un Wi-Fi - La taille de l'application. Réduire le poids du téléchargement à 5 Mo environ et réduire le poids de l'application afin qu'elle ne puisse pas prendre beaucoup d'espace de stockage dans le téléphone |
| 10 | Pensez-vous que 5 jours soient suffisants pour tester l’application et être capable de répondre au questionnaire MARS ? | - Oui 5 jours suffisent pour parcourir l'application et remplir le questionnaire - Non, parce que les personnels ont beaucoup d'activités en même temps et il faut plus de temps voir, même un mois, pour parcourir l'application et remplir le questionnaire |
| 11 | Avez-vous quelque chose à ajouter, n’importe quoi par rapport à l’application ? | - On devait faire et briefing sur les MTN à affection cutanée et sur l'application dans les formations sanitaires d'une manière régulière ou fréquemment on devrait organiser des formations sur les maladies tropicales négligées plus souvent - On doit utiliser un langage plus communautaire dans l'application, même dans les parties définition des termes tels que les signes et symptômes pour permettre l'utilisation de l'application par des personnes telles que des relais communautaires et d'autres personnes dans la communauté |

**Focus Group Session 5.**

| **Num** | **Questions** | **Réponses** |
| --- | --- | --- |
| **1** | Avez-vous déjà utilisé une autre application mobile de santé dans votre pratique quotidienne ? Si oui, laquelle ? | Gynécologie  Wikipédia médical  Manuel MSD ; qui traite les sujets médicaux  Les médicaments essentiels  Les infirmiers ; qui décrit les signes et symptômes, la prise en charge des maladies y compris l'anatomie humaine  NQS ; qui contient les données d'enquête sur les maladies des enfants et des femmes enceintes |
| **2** | Quelle est votre opinion générale de WHO SkinNTDs app ? D’après votre compréhension, qu’est-ce que cette application pourrait faire pour vous ? | L'application importante pour le diagnostic des maladies elle permet de connaître les maladies qu'on ne connaissait pas avant avec leur description et le traitement |
| 3 | Quelles sont les fonctionnalités que vous avez identifiées dans l’application ? | Les fonctionnalités diagnostics, signes et symptômes, les images des maladies sur la vie de face et la vue arrière de management et traitement des maladies elle est répartition mondiale de ces maladies |
| 4 | Quelles fonctionnalités pensez-vous essentielles à l’application ? Quelles sont celles que vous ne changeriez-pas. Pourquoi ? | Signes et symptômes et images qui permettent de préciser le diagnostic |
| 5 | Quelles fonctionnalités pensez-vous les moins essentielles ? Quelles sont celles que vous enlèveriez. Pourquoi ? | Les participants ont répondu de manière unanime ‘*’tout est bon’’* |
| 6 | Quelles fonctionnalités devraient absolument être dans l’application pour que vous l’utilisiez ou que vous la recommandiez ? | Les données par région (au Cameroun) concernant ces maladies  La possibilité d'agrandir des images pour mieux les visualiser  Une fonctionnalité pour la recherche à travers les images hors ligne  Insérer un code QR pour scanner les lésions sur le patient et envoyer dans l’application pour un feedback ou des rapports d'experts sur les images envoyées  La posologie dans la partie management ou prise en charge |
| 7 | Si vous deviez utiliser l’application ou si un personnel de sante en service dans une formation sanitaire devait utiliser l’application, quel serait le meilleur moment pour utiliser l’application ? | Le meilleur moment pour utiliser l'application c'est pendant la consultation lorsqu'on est en face du malade  À tout moment. « Il faut se familiariser avec l’application là à tout moment ».  Lorsqu'on est bloqué devant un cas particulier : « l’application est une sorte de formation, on doit lire cette application à tout moment. On va utiliser l’application lorsqu’on est bloqué, pour poser notre diagnostic. »  De préférence en continu même hors service pour pouvoir se familiariser à l'application et utiliser même lorsqu'on diagnostic. » est devant un patient sans utiliser son téléphone ou exploiter les informations qu'on a vu dans l'application lorsqu'on est devant un patient pour ne pas utiliser forcément le téléphone devant le patient  Pas devant le patient  « C’est mieux d’utiliser l’application en continu, il ne faut pas seulement être en formation sanitaire pour l’utiliser. On peut être en communauté et trouver un cas compliqué ». |
| 8 | Entrevoyez-vous que l’application puisse être intégrée comme un dispositif médical standard dans votre service ? Pourquoi ? | Oui elle doit être utilisée à tout moment.  Oui pour réduire les complications et éviter les pertes en termes de temps et de ressources  Oui pour que ces maladies ne soient plus qualifiées de négligées  Oui car ça va permettre de réduire le taux de ces maladies |
| 9 | Après avoir utilisé l’application, qui pensez-vous qui en soit l’utilisateur final idéal ? | Le dermatologue  Les consultant et les laborantins aussi  Tous les personnels de santé |
| 10 | Quelle serait la Meilleure façon de disséminer l’application ?  Comment proposeriez-vous de procéder pour que le maximum de personnes utilisateurs final idéal puissent être au courant de l’application et puisse l’installer ? | Par les liens de téléchargement dans le groupe WhatsApp des personnels de santé  Informer les utilisateurs à travers les partages d'expérience car cela permet de savoir même comment utiliser l'application et d'utiliser l'application efficacement.  « Partager des notifications quotidiennes. S’il y a amélioration dans une application, qu’on vous informe qu’il y a des mises à jour » |
| 11 | Quels défis entrevoyez-vous pour la dissémination, l’installation et l’utilisation de l’application ? Comment pensez-vous que ces défis puissent être adressés ? | Le problème de connexion et l'application est lourde et prend beaucoup d'espace de stockage dans le téléphone. Ce n'est pas tous les personnels qui ont un téléphone Android et certains personnels n’ont pas de téléphone. A la question comment palier à cela un participant a répondu ‘’vous connaissez !’’ Les autres ont ajouté l'OMS doit doter chaque FOSA ou chaque personnel d'un téléphone Android avec l'application installer dedans, avec carte Sim pour la connexion chaque mois  Il y a le problème d'électricité qui fait que par moment le téléphone est éteint l'on doit doter chaque FOSA de plaque solaire ou de power Bank pour charger le téléphone.  « *Il faudrait d’abord même former les personnels sur la dermatologie. Oui parce que pour connaitre même et classifier les signes et symptômes là ! Par exemple les bulles, les boutons, les pustules, tout ça la... on va classer ça comment ? il faut une formation pointue* ».  « *Vous pensez que même si on partage les liens de téléchargement là, si les gens ne sont pas formes, ils vont utiliser l’application* ? ». |
| 12 | Pensez-vous que 5 jours soient suffisants pour tester l’application et être capable de répondre au questionnaire MARS ? | 5 jours sont suffisants même en deux jours on peut tester l'application et répondre au questionnaire |
| 13 | Avez-vous quelque chose à ajouter, n’importe quoi par rapport à l’application ? | Ajouter des parties historique, prévention, et cause de ces pathologies et aussi former le personnel sur la dermatologie le dermatologue  Il faut réviser l'application et proposer des mises à jour comme on le fait pour d'autres applications sur Play store  Il y a beaucoup de personnes de peau blanche ce qui pourrait biaiser le diagnostic pour les malades de peau noire dans notre contexte. Ajouter plus d'image avec la peau noire  Les images doivent bien charger dans l'application pour être visibles.  Insister sur la formation. |

**Focus Group Session 6.**

| **Num** | **Questions** | **Réponses** |  |
| --- | --- | --- | --- |
| 1 | | Quelles fonctionnalités pensez-vous essentielles à l’application ? Quelles sont celles que vous ne changeriez-pas. Pourquoi ? | Signes et symptômes ; Diagnostic ; Comment gérer ; Glossaire  Signes et symptômes : ça nous aide à diagnostiquer une pathologie/ « c’est vraiment notre bord. » on ne peut pas diagnostiquer sans symptômes.  C’est une porte d’entrée qui nous permet à penser à une maladie.  SkinNTDs : nous somme dans les MTN. C’est celle-là qui nous permet les MTN de la peau. « C’est comme si ça sort presque la physiopathologie ».  Diagnostic : c’est très essentiel. Ça nous montre avec les images. Cela enlève le doute sur la confusion des pathologies. Parce que tu vois la personne, tu vois l’image.  Dans S&S, il y a l’anatomie et choisie chaque pathologie à partir des signes vus. Dans le diagnostic il faut connaitre d’abord la maladie (son nom) par contre dans S&S, on ne connait pas encore la maladie, mais on là où elle est localisée et comment elle se présente. |
| 2 | | Quelles fonctionnalités pensez-vous les moins essentielles ? Quelles sont celles que vous enlèveriez. Pourquoi ? | Index mondial : mais je ne m’intéresse pas forcement a ce qui se passe dans d’autre pays.  Faire la différence entre « important » et « essentiel ».  Glossaire : c’est peut-être un surplus qu’on a nous a donner. C’est juste venu renforcer notre connaissance.  « Si on ouvre glossaire et si on ouvre SkinNTDs Learning hub, on a plus d’information et définitions dans SkinNTDs Learning hub. » |
| 3 | | Quelles fonctionnalités devraient absolument être dans l’application pour que vous l’utilisiez ou que vous la recommandiez ? | Signes & Symptômes;  Diagnostic  Skin NTDs learning hub |
| 4 | | Entrevoyez-vous que l’application puisse être intégrée comme un dispositif médical standard dans votre service ? Pourquoi ? | S’il y avait la possibilité d’avoir un document qui liste pour toutes les MTN, les S&S, les images et comment gérer et poser ce document sur la table du consultant. Ce serait l’idéal. C’est mieux des planches.  L’application sur téléphone c’est aussi bon. Car on peut se déplacer et aller en communauté. |
| 5 | | Utilisez-vous une autre application mobile de santé dans votre pratique quotidienne ? Et vos collègues ? | Non |
| 6 | | Après avoir utilisé l’application, qui pensez-vous qui en soit l’utilisateur final idéal ? | Le consultant. Tout le monde qui consulte. |
| 7 | | Quelle serait la Meilleure façon de disséminer l’application ? | Atelier de formation.  Partager l’application dans des groupes.  Il serait important de former tous les responsables des formations sanitaires. Ainsi, ils vont former tout le personnel de leur formation sanitaire., |
| 8 | | Pensez-vous que 5 jours soient suffisants pour tester l’application et être capable de répondre au questionnaire MARS ? | Oui. |
| 9 | | Autres | Créer un petit budget pour primer toute personne qui va notifier un cas suspect de MTN a affection cutanée (UB, Lèpre, Pian.).  Briefer les ASC polyvalents sur la définition des cas. |
